# Highly Sensitive and Selective Gas Sensors Based on NiO/MnO_2_@NiO Nanosheets to Detect Allyl Mercaptan Gas Released by Humans under Psychological Stress

**DOI:** 10.1002/advs.202202442

**Published:** 2022-07-15

**Authors:** Chunyan Li, Pil Gyu Choi, Yoshitake Masuda

**Affiliations:** ^1^ National Institute of Advanced Industrial Science and Technology (AIST) 2266‐98 Anagahora, Shimoshidami, Moriyama Nagoya 463‐8560 Japan

**Keywords:** allyl mercaptan, gas sensor, MnO_2_@NiO, NiO porous nanosheets, p–p heterojunction

## Abstract

NiO nanosheets are synthesized in situ on gas sensor chips using a facile solvothermal method. These NiO nanosheets are then used as gas sensors to analyze allyl mercaptan (AM) gas, an exhaled biomarker of psychological stress. Additionally, MnO_2_ nanosheets are synthesized onto the surfaces of the NiO nanosheets to enhance the gas‐sensing performance. The gas‐sensing response of the NiO nanosheet sensor is higher than that of the MnO_2_@NiO nanosheet sensor. The response value can reach 56.69, when the NiO nanosheet sensor detects 40 ppm AM gas. Interestingly, a faster response time (115 s) is obtained when the MnO_2_@NiO nanosheet sensor is exposed to 40 ppm of AM gas. Moreover, the selectivity toward AM gas is about 17–37 times greater than those toward confounders. The mechanism of gas sensing and the factors contributing to the enhance gas response of the NiO and MnO_2_@NiO nanosheets are discussed. The products of AM gas oxidized by the gas sensor are identified by gas chromatography‐mass spectrometry (GC/MS). AM gas detection is an unprecedented application for semiconductor metal oxides. From a broader perspective, the developed sensors represent a new platform for the identification and monitoring of gases released by humans under psychological stress, which is increasing in modern life.

## Graphical abstract

This paper discusses the direct fabrication of NiO nanosheets on gas sensor chips via a facile solvothermal process and the synthesis MnO_2_ on its surface for modification. The NiO/MnO_2_@NiO gas sensors have high sensitivity and selectivity for detecting allyl mercaptan vapor, which releases by humans under psychological stress. People can easily identify psychological stress and to avoid various health problems.

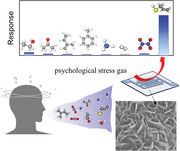

## 1 Introduction

Exhaled breath contains some gases in low concentrations,^[^ ^1^ ^]^ which can reflect physical conditions and thus serve as biomarkers of diseases.^[^ ^2^, ^3^ ^]^ Various exhaled gases have been acknowledged as biomarkers of diseases and metabolic processes, such as acetone gas for diabetes,^[^ ^4^ ^]^ isoprene for non‐alcoholic fatty liver disease,^[^ ^5^ ^]^ ammonia gas for renal disease,^[^ ^6^ ^]^ 1‐nonanal for lung cancer,^[^ ^7^ ^]^ and trimethylamine for uremia.^[^ ^8^ ^]^ According to the 2018 Report of Nihon Keizai Shimbun^[^ ^9^ ^]^ and a well‐known Japanese cosmetics company Shiseido Co., Ltd.,^[^ ^10^ ^]^ bad smells in the breath of people under psychological stress, often described as “onion smell,” are caused by the presence of AM and dimethyl trisulfide (DMTS) gas, and wrote the papers.^[^ ^11^ ^]^ Excessive psychological stress leads to various adverse effects, including nervousness, anxiety, irritability, depression, tension, and other negative emotions, thereby affecting one's sense of happiness. Long‐term continuous psychological stress may lead to health‐related problems by reducing immunity, by inducing various diseases, such as migraines, heart diseases, skin diseases, and cancer, and by affecting chronic diseases.^[^ ^12^ ^]^ Psychological stress is a major problem in modern life. Therefore, it is vital that psychological stress is identified and relieved to enable people to actively participate in daily work and life, to gain a sense of happiness, and to maintain good health. AM is a small allyl derivative containing a thiol group. It is a metabolite of garlic and some other allium plants. As a flavoring agent, AM has the ability to improve the flavor of food, and also can reduce cholesterol synthesis, and inhibit cancer.^[^ ^13^ ^]^ Although the GC/MS has been developed to detect and quantify AM gas,^[^ ^13^, ^14^ ^]^ metal oxide semiconductors (MOS) sensors for the detection of AM gas are currently unavailable.

Gas sensors based on MOS can noninvasively screen, analyze, and monitor various respiratory and systemic diseases by detecting breath signatures. Conventional diagnostic methods, such as blood measurements, cytology, and radiography (e.g., computed tomography) are invasive, highly uncomfortable, and do not support a wide population range. Non‐invasive diagnostic methods based on chemical sensors that detect breath signatures can offer several advantages, including high sensitivity, good stability, miniaturization, low power consumption, simple structure, and cost savings.^[^ ^15^, ^16^, ^17^ ^]^ These factors contribute to the massive need for non‐invasive technologies based on MOS sensors.

Among the known MOS materials, NiO, a p‐type semiconductor, has been extensively studied because of its wide bandgap energy in the range of 3.6–4.0 eV.^[^ ^18^ ^]^ Moreover, NiO has various advantages, including outstanding chemical and thermal stabilities, cost effectiveness, environmental benignity, and transportation properties that are promising for gas sensing.^[^ ^19^, ^20^ ^]^ Gas sensors based on NiO with different morphologies have been developed; examples include ultrathin NiO nanosheets to detect ethanol,^[^ ^21^ ^]^ hierarchical NiO hollow microspheres to detect n‐butanol,^[^ ^22^ ^]^ and NiO nanowires to detect ammonia.^[^ ^23^ ^]^ Notably, gas sensors based on pristine NiO without effective modification are ineffective at achieving excellent comprehensive performance, namely sensitivity, selectivity, and rapid response at an optimum operating temperature.^[^ ^24^ ^]^ In addition to increasing the specific surface area of NiO through enhancing surface morphology, as mentioned above, the performance of MOS gas sensors can be improved by using noble metal catalysts^[^ ^25^, ^26^ ^]^ and by constructing heterostructures using metal oxides.^[^ ^27^, ^28^ ^]^ The construction of heterostructures between two semiconductors has been demonstrated as an efficient method to improve gas sensing performance. Heterostructures with vigorous interactions between heterointerfaces greatly affect oxygen adsorption, catalytic activity, and charge transfer,^[^ ^29^ ^]^ which further enhances the sensing response. We designed the materials with p–p heterojunction in consideration. Compared with n‐type semiconductor gas sensors, p‐type semiconductor gas sensors have lower resistance values and lower operating temperatures. However, the sensitivity of p‐type metal oxide gas sensors is low. By forming nanocomposites with other semiconductors, the performance of gas sensors can be effectively improved. The composite of different nanomaterials can effectively control the carrier concentration and can promote the adsorption of target gas and oxygen, adjust the resistance change during gas adsorption, which can effectively improve the gas sensing performance. Various gas sensors based on p–n heterojunctions have been studied over the past few decades, but there are few studies on gas sensors of p–p heterojunction. Because of its low cost, high chemical stability, natural abundance, and low toxicity,^[^ ^30^ ^]^ the semiconductor MnO_2_ has been applied to supercapacitors,^[^ ^31^, ^32^ ^]^ rechargeable batteries,^[^ ^33^, ^34^ ^]^ and catalysts.^[^ ^35^ ^]^ However, MnO_2_ has received very limited attention for gas sensing because of its low sensing response. Several nanostructures of MnO_2_ (including nanowires, nanograins, hierarchical hollow nanospheres, and nanorods) have been developed for the detection of triethylamine, hydrogen, ammonia, and ethanol.^[^ ^36^, ^37^, ^38^, ^39^ ^]^ Recently, some literature has demonstrated that the gas‐sensing performance of MnO_2_ was improved by applying heterostructure formation techniques. For example, ZnO nanoparticles combined with MnO_2_ exhibited higher formaldehyde‐sensing performance.^[^ ^40^ ^]^ Moreover, Sharma et al. fabricated halloysite nanotubes using Fe_3_O_4_ and 3D MnO_2_ nanoflakes to detect acetone gas with a sensitivity of 36.5 for 100 ppm at 150 °C.^[^ ^41^ ^]^ However, to the best of our knowledge, MnO_2_@NiO has not been used for other gas detecting, in addition to being used for NOx detection.^[^ ^42^ ^]^ Therefore, it is very promising to study the gas sensitivity of MnO_2_@NiO nanocomposites.

In this study, we used NiO nanosheets and MnO_2_‐modified NiO nanosheets to detect AM gas. The NiO porous nanosheets were synthesized via a facile solvothermal reaction at low temperature (<100 °C), and the MnO_2_ nanosheets were prepared via an etching–recrystallization process at low temperature (65 °C). Thus, we successfully coated the NiO nanosheets with MnO_2_ nanoparticles to form a well‐defined p–p heterojunction structure. After calcination, which removed water and intercalated impurities, NiO exhibited a porous structure. The structure and gas‐sensing performance of the prepared products were characterized. Because of its porous structure, high adsorbed oxygen content, and low activation energy, the NiO‐based gas sensor was highly sensitivity toward AM gas with an ultralow detection limit of 15 ppb. Coating MnO_2_ onto the NiO nanosheet surfaces accelerated the response speed. Finally, AM gas selectivity in the presence of typical confounders (i.e., isoprene, ethanol, acetone, toluene, hydrogen, ammonia, and nitrogen dioxide) during breath monitoring was evaluated. We believe that the NiO and MnO_2_@NiO gas sensor, fabricated using a low‐cost and mild synthesis method, has tremendous potential in the future because of its excellent performance in detecting AM gas. Notably, to our knowledge, MOS gas sensors have not been used for the detection of AM gas.

## 2 Results and Discussion

### 2.1 Characterization of NiO and MnO_2_@NiO

**Figure 1:**
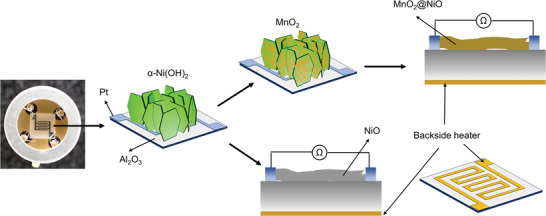
Schematic of the in situ synthesis the NiO and MnO_2_@NiO nanosheets on Al_2_O_3_ sensor chips.

An illustration of the synthesis procedure is shown in **Figure** **1**. The Ni(OH)_2_ nanosheets were grown in situ on the alumina chip via a chemical bath deposition method.

**Figure 2:**
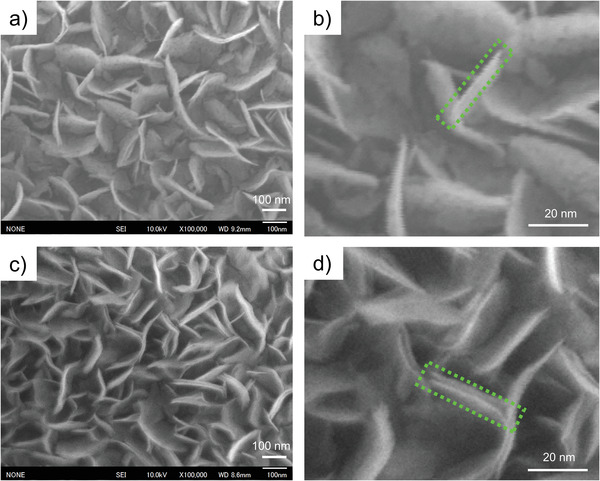
SEM images: a) NiO nanosheets, b) Magnified image of the NiO nanosheets, c) MnO_2_@NiO nanosheets, and d) Magnified image of the MnO_2_@NiO nanosheets.

The top‐view Field emission scanning electron microscopy (FE‐SEM) images of the NiO nanosheets under low and high magnifications are shown in **Figure** **2**a–d. As shown in Figure 2a, the NiO nanosheet arrays were vertically aligned on the substrate. Under high magnification (Figure 2b), the obtained NiO nanosheets exhibited a fine nanosheet structure; each nanosheet was assembled from numerous nanoparticles to produce a mesoporous structure. The average thickness and length of an individual sheet in NiO were approximately 21.7 and 150 nm, respectively. The distribution of the sheet thickness was in the range of 15–28 nm (Figure 2b). This porous film morphology is attractive for gas sensing because the analytes can easily penetrate the film and interact with its large surface. To preserve this sensor‐favorable film architecture, NiO nanosheets with attached MnO_2_ nanosheets were fabricated using only KMnO_4_ on the Ni(OH)_2_ film via a facile process at moderate temperatures. The obtained nanocomposites were calcined in air, thus transforming MnO_2_@Ni(OH)_2_ into MnO_2_@NiO. Water and inserted ions were removed by further calcination, and NiO nanosheets with attached MnO_2_ were obtained. As shown in Figure 2c,d, the overall morphology of the vertically aligned nanosheets did not change after the low‐temperature treatment used to synthesize MnO_2_ onto the Ni(OH)_2_ nanosheets. Moreover, the self‐standing feature and nanosheet morphology were well retained after calcination at 400 °C. Only parallel sheets (nanosheet layers) were generated near the original nanosheets. Because during the slow corrosion process, only a small fraction of the surface layer was corroded, the nanosheets structure of Ni(OH)_2_ does not be seriously destroyed. Then the generated MnO_2_ and Ni(OH)_2_ recovered the remaining nanosheets. The formation of the parallel layer is probably by the formation of tiny grooves when KMnO_4_ corrodes Ni(OH)_2_.^[^ ^43^ ^]^ The formation of such parallel sheets can expose more active edge sites, which could supply more adsorption and reactive sites to enhance the sensing performance. However, the thickness of the NiO nanosheets decreased to approximately 8.9 nm. In addition, MnO_2_ nanosheets with a thickness of approximately 11.5 nm were obtained when the synthesis time was 24 h (Figure S1, Supporting Information), and the MnO_2_ nanosheets were tightly attached to the NiO nanostructure.

The MnO_2_ growth process can be considered a coordinated etching and recrystallization process. During this process, KMnO_4_ oxidizes Ni(II) to form Ni(III), but this reaction proceeds very slowly under neutral conditions. The reaction can releases OH^−^ to facilitate the etching of Ni(OH)_2_. The generated Ni(III) can simultaneously oxidize water and reduce itself to nickel(II); this corresponds to a recrystallization process. This etching and recrystallization process includes the following reactions:

(1) $${\mathrm{KMnO}}_{4}+3\mathrm{Ni}{\left(\mathrm{OH}\right)}_{2}\to {\mathrm{MnO}}_{2}+3\mathrm{NiOOH}+\mathrm{KOH}+H_{2}O$$

(2) $$4\mathrm{NiOOH}+2H_{2}O\to 4\mathrm{Ni}{\left(\mathrm{OH}\right)}_{2}+O_{2}\;$$

This process is consistent with the experimental results because it was difficult to generate MnO_2_ in a substrate without the Ni(OH)_2_ film or in solution.

The low magnification and cross‐sectional SEM images (Figure S2a–d, Supporting Information) provided a full view of the structure of the sensor chips. In particular, the MnO_2_ nanosheets were uniformly distributed on NiO and were tightly connected to NiO.

**Figure 3:**
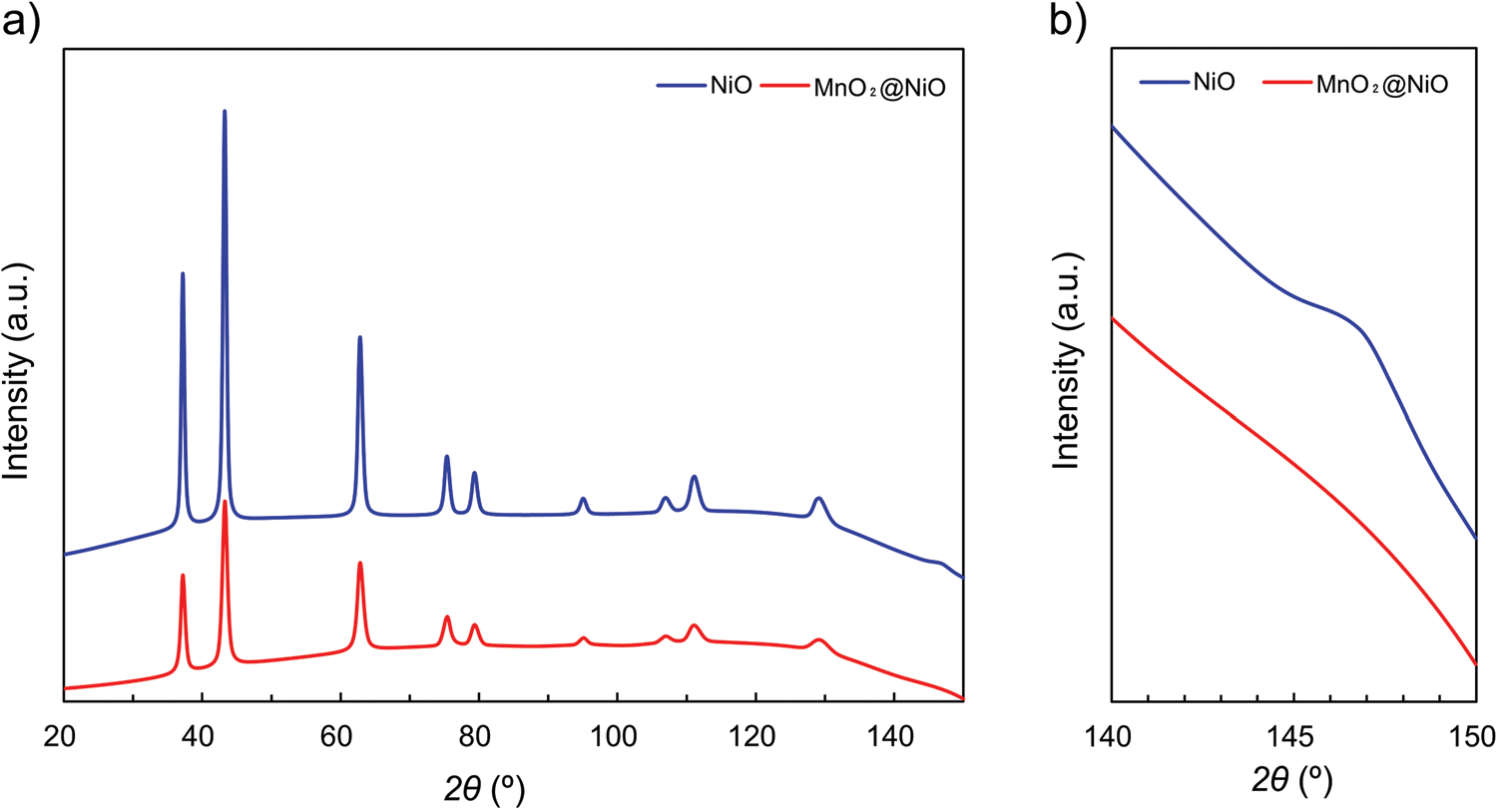
a) XRD spectra of NiO (blue) and MnO_2_@NiO (red). b) Enlarged spectra in the 2*θ* range of 140°–150°.

To further investigate the crystal structure, X‐ray diffraction (XRD) patterns of the samples were collected. The NiO and MnO_2_@NiO powders were characterized directly by using XRD. The obtained XRD patterns are shown in **Figure** **3**a, and the red line represents the XRD pattern of NiO. All the diffraction peaks of the NiO pattern were indexed to the cubic phase (JPCDS Card No. 65‐2901). The XRD pattern of the MnO_2_@NiO nanosheet arrays, shown in Figure 3a (blue line), was indexed to the cubic phase (Mn_0.66_Ni_0.34_O) (JPCDS Card No. 81‐9152). The XRD patterns of NiO and MnO_2_@NiO were similar. However, peaks corresponding to MnO_2_ were absent in the pattern because of the formation of Mn_0.66_Ni_0.34_O. Nevertheless, a small peak at 147° assigned to the (511) reflection of NiO was detected, as shown in Figure 3b. Thus, the NiO and MnO_2_@NiO phases were distinguishable. Obviously, the peak of MnO_2_@NiO becomes lower and broader than the pure NiO, indicating that the crystallization degree was larger in NiO. The average crystallite size was calculated using the Scherrer equation. Using the (111), (200), and (220) lattice planes, the average crystallite sizes were respectively 15.47, 14.15, and 15.3 nm for NiO and 11.67, 10.19, and 9.14 nm for MnO_2_@NiO (Figure S3, Supporting Information). The crystallites in the MnO_2_@NiO composite were smaller than those in the NiO nanosheets, which was consistent with the SEM results.

**Figure 4:**
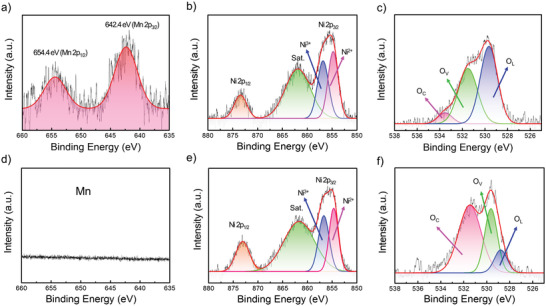
High‐resolution Mn 2p, Ni 2p, and O 1s XPS spectra of a–c) MnO_2_@NiO and d–f) NiO.

X‐ray photoelectron spectra (XPS) were used to investigate the chemical compositions and metal oxidation states of the NiO and MnO_2_@NiO nanosheets. The XPS spectra of the NiO and MnO_2_@NiO materials are shown in **Figure** **4**. The C 1s peak at 284.6 eV was attributed to the reference element used for calibration. The Mn 2p spectrum of MnO_2_@NiO contained two distinct peaks located at 642.4 and 654.4 eV, corresponding to the Mn 2p_3/2_ and Mn 2p_1/2_ signals, respectively. The spin‐energy separation was 12.2 eV, which further confirmed that the Mn element was in the Mn^4+^ state (Figure 4a). In contrast, there were no Mn peaks in the Mn 2p spectrum of NiO (Figure 4d). Therefore, MnO_2_ was successfully deposited on the surface of NiO. In the Ni 2p spectrum, there were two spin‐orbit doublets centered at 873.2 and 855.5 eV, which were assigned to the Ni 2p_1/2_ and Ni 2p_3/2_ signals of NiO, respectively (Figure 4b,e). Therefore, the Ni ion existed in the composites. The spin‐energy separation between the two Ni 2p peaks was approximately 17.7 eV, which matched the previously reported values of NiO.^[^ ^44^ ^]^ Additionally, the O 1s spectra of the nanosheets clearly demonstrated a variation in the chemical states of the oxygen atom (Figure 4c,f). The spectra were deconvoluted into three single peaks, corresponding to chemisorbed oxygen (O_C_), deficient oxygen (O_V_), and crystal lattice oxygen (O_L_) arising from the Ni‐O and Mn‐O bonds. The relative percentage of O_L_ in MnO_2_@NiO was 52.3%, which was greater than that in NiO (11.2%). This huge difference is due to the oxygen in MnO_2_ lattice; and suggested that a good interface existed between the MnO_2_ and NiO phases, in agreement with the XRD result. According to a quantitative analysis of the XPS spectra, the atomic ratio of Mn:Ni was approximately 89% in the surface layer by the photoionization cross‐sectional area of the element.

The mass content of MnO_2_ in the MnO_2_@NiO composite was determined by using TGA. In contrast to the XPS analysis, the MnO_2_ content in the entire composite material, not just in the surface layer, was determined using TGA. Figure S4 (Supporting Information) shows the representative TGA curves of the NiO and MnO_2_@NiO nanosheets obtained in air. For comparison, we also measured the TGA curve of the sample synthesized for 4 h. These samples showed a slight weight loss of 6 wt% below 265 °C, which was ascribed to the evaporation of adsorbed water and ethanol molecules. The weight loss within the temperature range of 265–307 °C was attributed to the decomposition of Ni(OH)_2_ into NiO. As shown in Figure S4 (Supporting Information), the weight loss from the decomposition of Ni(OH)_2_ was the largest. For samples containing MnO_2_, the weight loss was smaller because the sample quality did not change as the temperature increased for MnO_2_. Therefore, the composite material comprising MnO_2_ and Ni(OH)_2_ underwent a smaller weight change than the samples containing only Ni(OH)_2_. Moreover, the weight change decreased with an increase in the MnO_2_ content (i.e., increase in synthesis time for MnO_2_). From the residual weight of MnO_2_@Ni(OH)_2_, the mass content of MnO_2_ in the composite was calculated, which was 9.77 wt% after 2 h and 20.43 wt% after 4 h of synthesis.

### 2.2 Temperature Effects on AM Sensing

**Figure 5:**
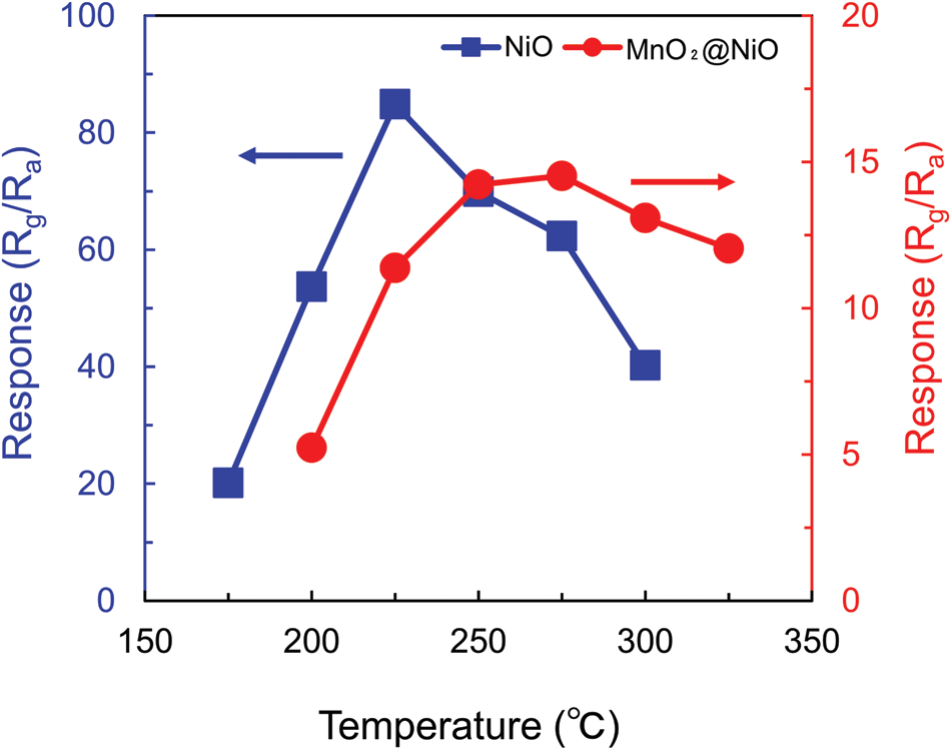
Response of the gas sensors exposed to 30 ppm of allyl mercaptan gas at different operating temperatures.

The operating temperature is an important parameter of gas sensors based on chemiresistors. The temperature‐dependent properties of the NiO and MnO_2_@NiO nanosheets were determined over the temperature range of 175–325 °C. In particular, the responses of the nanosheets exposed to 30 ppm of AM vapor in air were measured at different operating temperatures (**Figure** **5**). The operating temperature clearly played an important role in the sensor response. The measured response first increased, then reached a maximum, and finally decreased with an increase in temperature. Both gas sensors displayed a volcano‐shaped correlation between gas response and operating temperature. This temperature dependence arises from the chemical interactions between the sensor and the tested gas.^[^ ^45^ ^]^

As the operating temperature increases, absorbed molecular oxygen generates more active oxygen species, namely O_2_^−^, O^−^, and O^2−^,^[^ ^46^, ^47^ ^]^ and this reaction is maintained until the optimum operating temperature is reached, thus increasing the response. Moreover, the tested gas does not possess sufficient thermal energy to react with oxygen ions at low temperatures. As the temperature is further increased, the desorption of oxygen ion species becomes predominant owing to the higher activation energy;^[^ ^48^ ^]^ Simultaneously, desorption of the adsorbed gas molecules is promoted which leads to the reaction time is insufficient. Therefore, insufficient reaction times lead to lower responses toward the target gas, AM. Thus, the optimum operating temperature is a balance point between two conflicting mechanisms. The optimum operating temperatures and corresponding responses (*S*) were respectively 225 °C and 53 for NiO, 275 °C and 10.8 for MnO_2_@NiO. Compared with the MnO_2_@NiO sensor, the optimum operating temperature of the NiO sensor was lower by 50 °C, which was caused by the large surface area and low activation energy.^[^ ^49^ ^]^ All the measurements thereafter were conducted at 225 °C for NiO and 275 °C for MnO_2_@NiO.

### 2.3 AM Sensing Performance

**Figure 6:**
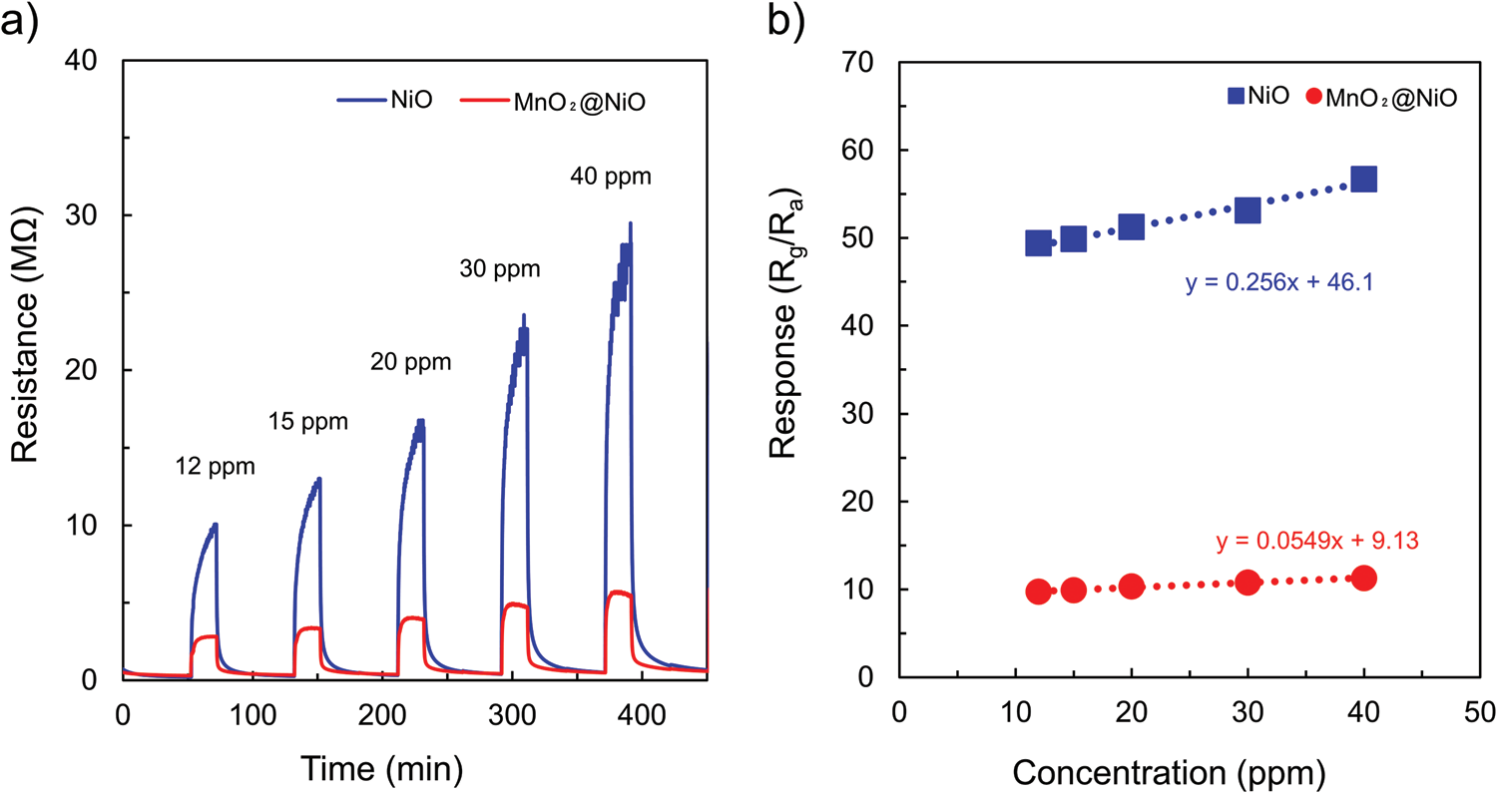
a) Dynamic curve of the gas sensors exposed to different allyl mercaptan concentrations at the optimal temperature of NiO and MnO_2_@NiO. b) Linear fit of the responses as a function of allyl mercaptan concentration at optimal temperatures of NiO and MnO_2_@NiO.

Before the tested gas was exposed to the sensing material, the gas sensor was allowed to stabilize at each operating temperature in fresh air for 2 h. The NiO and MnO_2_@NiO sensors were exposed to AM vapor with concentrations of 12, 15, 20, 30, and 40 ppm at their optimum operating temperatures, and the resulting variations in resistance as a function of time were recorded (**Figure** **6**a). The resistance of the NiO gas sensor increased significantly upon exposure to AM vapor. When the NiO gas sensor was exposed to 40 ppm of AM vapor, the resistance increased from 482 kΩ to 29.5 MΩ, corresponding to a response of 56.69. Furthermore, the resistance increased with an increase in the concentration of AM vapor. The response increased monotonically from 49.39 at 12 ppm of AM vapor to 56.69 at 40 ppm of AM vapor, as shown in Figure 6b (blue line). Therefore, the response of the NiO gas sensor is dependent on the concentration of AM vapor. The MnO_2_@NiO sensor exhibited an inferior response of 9.73 and 11.28 at 12 ppm and 40 ppm of AM vapor, respectively.

Repeatability is another important parameter of sensors from the perspective of practical applications. This parameter was investigated by successively subjecting the MnO_2_@NiO sensor to four cycles of gas‐sensing testing. Figure S5a (Supporting Information) shows the typical dynamic curves for four successive cycles of testing in which the MnO_2_@NiO sensor was exposed to 12 ppm of AM vapor under the same conditions. As shown in Figure S5b (Supporting Information), the gas sensor retained the initial response amplitude after four cycles, irrespective of the time taken to reach stability (1 or 5 h). Moreover, the response value, response time, and recovery time were almost reproducible. Therefore, the reversibility and repeatability of the sensor were satisfactory for the detection of AM gas.

Figure S6a (Supporting Information) shows the dynamic curves of the NiO and MnO_2_@NiO sensors to 20 ppm AM gas within 30 d. The change in resistance of baseline can be obtained before the sample gas enters the sensor chambers. The baseline resistance values of the two sensors increase over time, after 30 d the resistance of NiO sensor was 2.7 times to that of the initial value and this increase amplitude is a little larger than that of MnO_2_@NiO sensors. However, the resistance of the corresponding response increasing, therefore, the impact of baseline resistance change on the stability of the sensor can be ignored within 30 d. Figure S6b (Supporting Information) shows the response values of 20 ppm allyl mercaptan in 30 d. Comparing with NiO sensors, which response values decreased about 28% during the 30 d, those of MnO_2_@NiO sensors to 20 ppm AM varied in small ranges without significant reduction. Based on the response value in the initial detection, the maximum fluctuation is only ±8% in the detection period.

**Figure 7:**
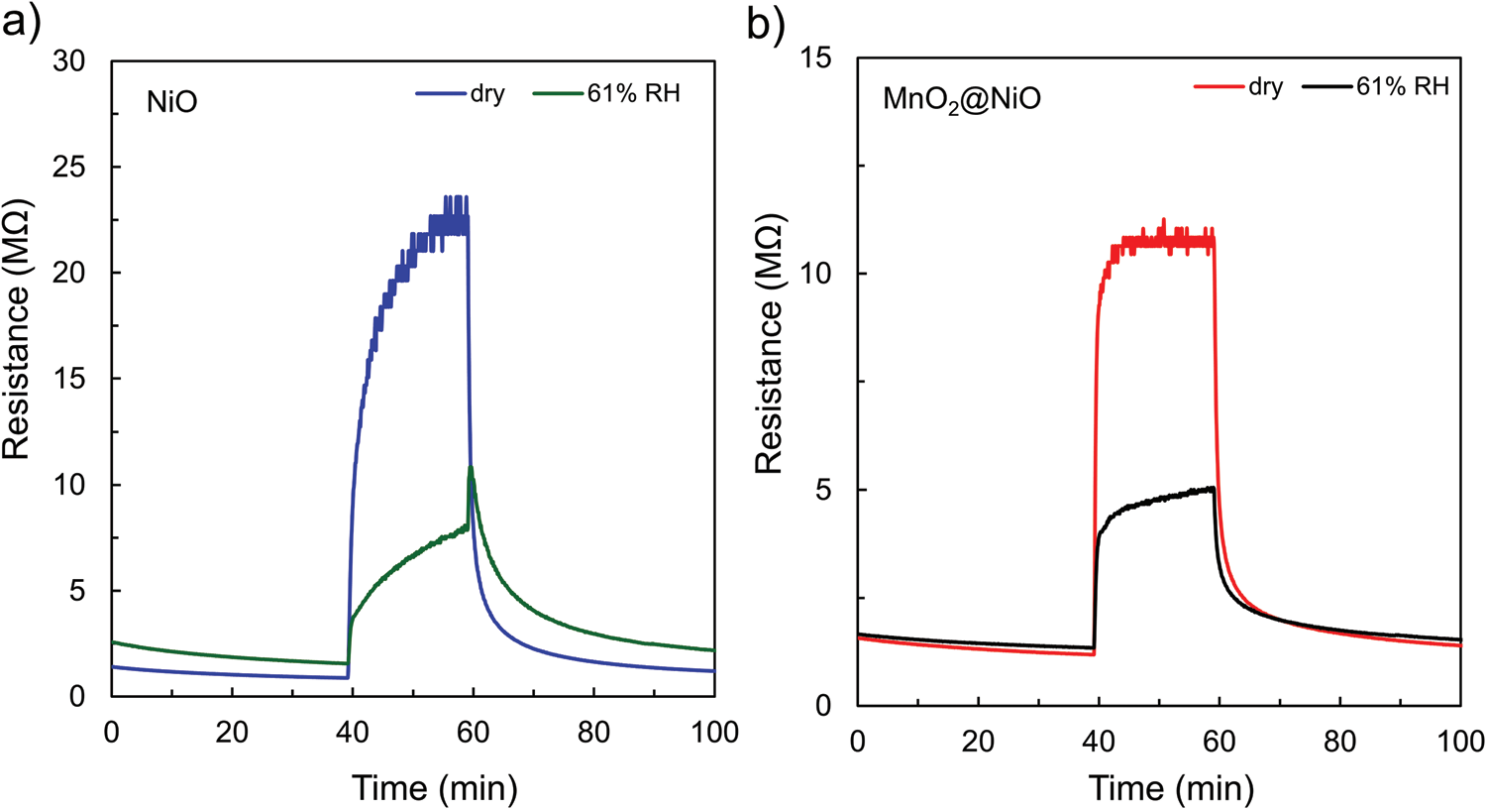
Dynamic sensing response curves under different relative humidity: a) NiO gas sensor and b) MnO_2_@NiO gas sensor.

Humidity is a critical interfering factor in gases released by human body. The H_2_O molecule will occupy more active sites, leading to the deterioration response to the target gas. To study the effect of humidity on the sensing performance of the NiO and MnO_2_@NiO sensors, 12 ppm AM target gas under a relative humidity (RH) of 0% (dry air) and 61% (73% RH air was employed as buffer gas to modulate the 75 ppm AM gas) were detected. **Figure** **7**a,b shows the dynamic curves under a different relative humidity of NiO and MnO_2_@NiO sensors, the responses of NiO sensor were 26.21 and 5.32 under 0% and 61% RH; the values were 9.73 and 3.74 for MnO_2_@NiO sensor. Obviously, the response of the MnO_2_@NiO sensor under 61% RH only about 40% reduction compared to this value in dry air, which shows a lower effect of RH. In addition, the responses of AM gas were carried out under different RH (34–61%) as shown in Figure S7a (Supporting Information). The different RH can only be adjusted by changing the gas flow rate, i.e., gas concentration, due to instrument limitations. The sensor response increased with increasing AM gas concentration at 225 °C, with favorable linearity being observed under the humid conditions. The difference between the gas responses obtained under the dry and humid conditions was smaller in the MnO_2_@NiO sensor (40%) than in the pure NiO sensor (20%), regardless of humidity change, as shown in Figure S7b (Supporting Information). These results indicate that the real concentration of AM gas can be calculated by detecting the response value of AM gas in humid air, which suggests a high probability for use in detection of the AM gas released by humans.

The response time (*τ*_res_) and recovery time (*τ*_rec_) are closely related to the detection speed of the sensor, and they are two important parameters for the evaluation of gas sensing performance. The dynamic response characteristics of the sensors exposed to AM vapor were investigated. The NiO and MnO_2_@NiO sensors were exposed to AM gas, and the resulting variations in electrical resistance as a function of time were recorded (Figure S8a,b, Supporting Information). The resistance of the NiO sensor varied over a long time after the sensor was exposed to AM vapor, and the sensor response was slow. The *τ*_res_ and *τ*_rec_ values of the NiO sensor were respectively 480 s and 15 s at 40 ppm of AM vapor. However, the resistance of the MnO_2_@NiO sensor increased sharply at the initial stage and became constant. The *τ*_res_ and *τ*_rec_ values of the MnO_2_@NiO sensor were respectively 115 and 25 s at 40 ppm of AM vapor. Therefore, the MnO_2_@NiO sensor responded faster than the NiO sensor, but the recovery speeds of the two sensors were similar. In the interest of deeper investigation of the responding and recovering characteristics of the gas sensors based on NiO and MnO_2_@NiO nanosheets, the response and recovery time of the gas sensor to different concentrations of AM were measured under the optimal temperatures, and the results are shown in Figure S9 (Supporting Information). The chemical activities of the semiconductor oxide sensors can be fully excited at the optimum operating temperature. Both the response and recovery times decreased with an increase in the temperature, which was likely caused by the enhanced electron transition between the conductor band and surface Fermi level during gas reactions, adsorption/desorption processes, and diffusion rate. Therefore, the response time of the MnO_2_@NiO sensor was faster than that of the NiO sensor, which was attributed to the higher optimum temperature (275 °C) of the MnO_2_@NiO sensor. Furthermore, the smaller crystallite size (calculated by using the Scherrer equation; Section 2.1) of the composite can offer a larger active surface area for fast adsorption. This may be another reason for the rapid response of the MnO_2_@NiO sensor. As well as the formation of p–p heterojunctions can shorten the response time, it is elaborated in Section 2.6.

**Table** **1** compares the sensor performance of different structures of NiO (as far as I know, no one has ever used MnO_2_@NiO gas sensor to detect sulfur‐containing gas) gas sensor for detection of sulfur‐containing gas in literature. Since no one has yet used the MOS sensors to detect AM gas, gas compounds including sulfur can only be selected for comparison. The results clearly show that the gas sensor containing NiO has superior detection performance for sulfur‐containing gases. This demonstrates that the NiO and MnO_2_@NiO sensors have potential to detect AM gases in practical applications.

**Table 1:** Comparison of sulfur‐containing gas sensing performance among gas sensors based on NiO materials

Sensing materials	Temperature [°C]	Gas	Response	Concentration [ppm]	Refs.
NiO microflowers	RT	H_2_S	8.8	97	^[^ ^50^ ^]^
NiO spheres and hexagonal	75	H_2_S	74	60	^[^ ^51^ ^]^
NiO meshed nanowalls	50	H_2_S	137.3	100	^[^ ^52^ ^]^
porous NiO microspheres	200	H_2_S	27.2	20	^[^ ^53^ ^]^
CuO/NiO nanowall	133	H_2_S	36.9	5	^[^ ^54^ ^]^
NiO/ZnO nanowire	RT	H_2_S	536	1000	^[^ ^55^ ^]^
CuO‐NiO core–shell microspheres	260	H_2_S	47.6	100	^[^ ^56^ ^]^
Fe_2_O_3_ nanoparticle decorated NiO nanoplates	300	H_2_S	26.55	200	^[^ ^57^ ^]^
NiO nanowalls	300	SO_2_	8.8%	20	^[^ ^58^ ^]^
NiO‐ZnO nanodisks	240	SO_2_	16.25	20	^[^ ^59^ ^]^
Porous NiO nanosheets	225	AM	56.7	40	Present work
MnO_2_@NiO nanosheets	275	AM	9.7	12	Present work

### 2.4 LOD of the Gas Sensors

The limit of detection (LOD) is a key figure of merit in chemical sensing. At the LOD concentration, an analytical signal can be differentiated clearly from noise. In accordance with the IUPAC procedure, the LOD of a sensor can be obtained when the noise level is lower than one third of the signal level. It is typically defined as the analyte concentration that causes a response 3 times higher than the noise level of the device (i.e., in the absence of the analyte).^[^ ^60^, ^61^ ^]^ The theoretical LOD can be calculated using Equation (3):

(3) LOD=3×RMS/S

where RMS is the standard root‐mean‐square deviation for the noise of the device and *S* is analytical sensitivity. The RMS values were determined using the baseline fluctuations of the blank sample. The initial resistance was calculated from the 100 data points before the AM signal was detected, and the *RMS* was derived from the baseline. As shown in Figure S10 (Supporting Information), for the NiO sensor, the RMS value of Δ*R*/*R*_air_ baseline noise (where *R*_air_ is the resistance of the device established under air flow before AM exposure and Δ*R* is a resistance change caused upon AM exposure) was 0.0013, giving a calculated LOD of 3 × 0.0013/0.2555≈0.015 ppm. For comparison, the LOD of MnO_2_@NiO was also calculated. The RMS value of *ΔR*/*R*_air_ baseline noise was approximately 0.0006, resulting in a detection limit of 3 × 0.0006/0.0549≈0.032 ppm.

Another simple method is the direct calculation of the signal‐to‐noise‐ratio (SNR). SNR needs to be greater than 3 to calculate the LOD. As shown in Figure 6a, AM gas was detectable at concentrations down to 12 ppm (the lowest AM concentration provided by our current experimental setup), and the AM signal was clearly distinguishable from the baseline. The huge SNR (>5.6 × 10^4^) enabled the detection of even lower concentrations of AM gas with an LOD of 0.65 ppb, which was calculated using the typical SNR of 3. AM is a new gas detectable by metal oxides. The calculated sensitivity of the NiO sensor was 0.2555 ppm^−1^, indicating that the sensitivity of this gas sensor was good. It is noteworthy that the theoretical LOD of the NiO sensor was as low as 15 ppb and 0.65 ppb using two different methods. Although these two values are different, they are sufficiently low to detect AM. Therefore, this sensor can be applied to detect the low concentration of gas generated by the human body under stress.

### 2.5 Selectivity

**Figure 8:**
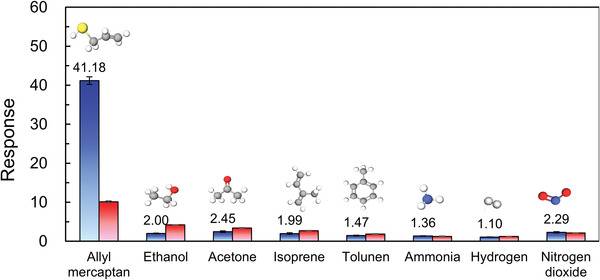
Responses of the NiO and MnO_2_@NiO sensors exposed to 20 ppm of various gases to test selectivity.

In practical applications, gas sensors must operate in complex environments containing various gases. Therefore, high selectivity is an important characteristic of reliable sensors because poor selectivity can lead to false alarms. The selectivity of the sensors was comprehensively and accurately assessed. The sensors were exposed to several interfering gases, namely ethanol, acetone, isoprene, toluene, ammonia, hydrogen, and nitrogen dioxide, at a constant gas concentration of 20 ppm (**Figure** **8**), and the responses of the NiO and MnO_2_@NiO sensors were measured at the optimum operating temperatures of 225 °C and 275 °C, respectively. Thus, the sensor with the best response and selectivity characteristics was determined. The selectivity and response of both sensors were higher toward gaseous AM than the other tested gases. The responses of the NiO sensor were 41.18, 2.00, 2.45, 1.99, 1.47, 1.36, 1.10, and 2.29 toward AM, ethanol, acetone, isoprene, toluene, ammonia, hydrogen and nitrogen dioxide, respectively. Most remarkably, the response of the NiO‐based sensor toward AM gas was at least 17 times larger than its response toward the other interfering gases. Moreover, the responses of the NiO sensor were similar with the MnO_2_@NiO sensor toward the interfering gases. The response of the NiO sensor was approximately 4 times higher than that of the MnO_2_@NiO sensor toward gaseous AM. Therefore, the selectivity of the NiO nanosheets is higher toward gaseous AM than MnO_2_@NiO nanosheets sensor.

The exact mechanism underlying the enhanced selectivity of the NiO sensor toward AM is not certain. However, it is well known that the selectivity of sensors toward tested gases is related to the surface structure and electronic characteristics of the sensor material, the reactions involving the target gas, and the adsorption capacity of the sensor material for the target gas. In this study, the observed selectivity was mainly ascribed to the bond energy (BE) of AM. IUPAC defines the BE as the average value of the gas‐phase bond‐dissociation energy.^[^ ^62^ ^]^ The BE of C‐S in AM (272 kJ mol^−1^) is significantly lower than those of the other molecules.^[^ ^63^, ^64^ ^]^ In a typical gas‐sensing mechanism, the gas is oxidized by the adsorbed oxygen species on the surface of the sensor. A low BE leads to facile bond cleavage, and the generated ions then participate in reactions with surface‐adsorbed oxygen species. According to the mass spectra of NIST, the most prone to ion generation of ethanol, acetone, isoprene, toluene, ammonia, hydrogen, and nitrogen dioxide are C‐C, C‐C, C‐H, C‐H, N‐H, H‐H, and N‐O. The BEs of ethanol (C‐C), acetone (C‐C), isoprene (C‐H), toluene (C‐H), ammonia (N‐H), hydrogen (H‐H), and nitrogen dioxide (N‐O) are respectively 346, 346, 411, 411, 386, 432 and 469 kJ mol^−1^ (Figure S11, Supporting Information). Because of the low C‐S BE, the reaction between the AM molecules and the adsorbed oxygen species can occur easily, which is expected to contribute to the high response of the NiO and MnO_2_@NiO nanosheet sensors.

### 2.6 Mechanism

**Figure 9:**
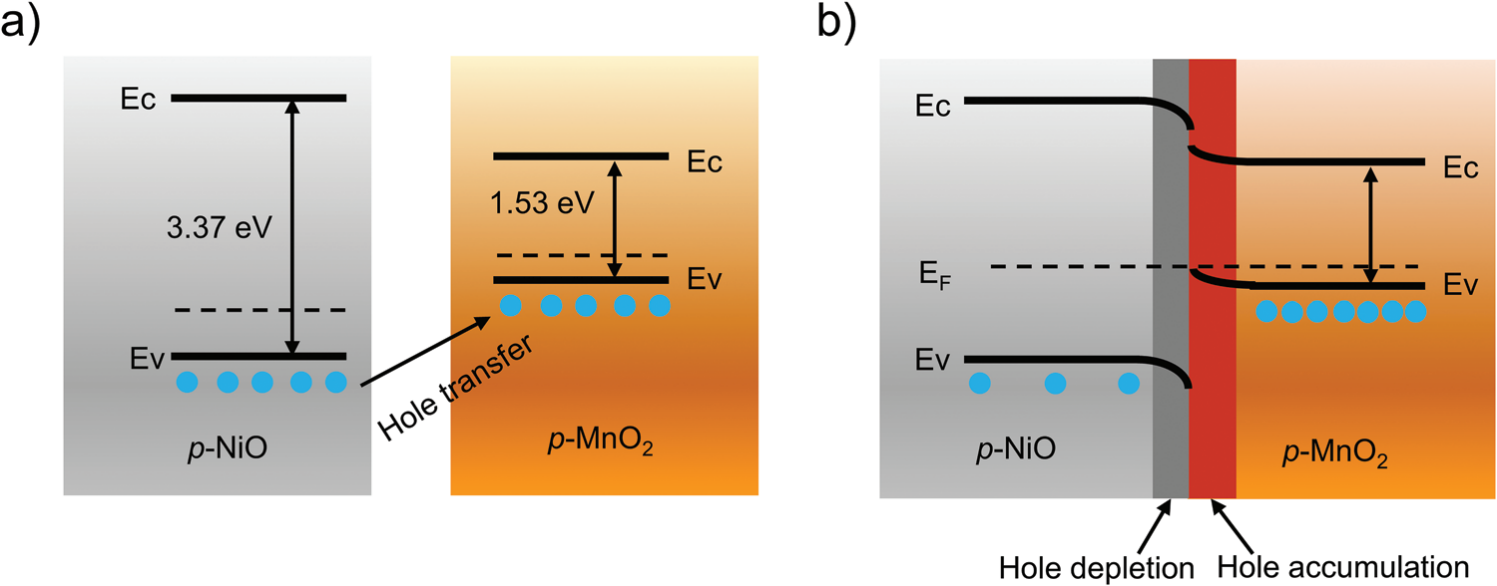
Energy band diagrams of NiO and MnO_2_ a) before and b) after contact. (*E*_F_ is Fermi energy level, *E*_C_ is the bottom of the conduction band, and *E*_V_ is the top of the valence band.).

In this section, we discuss the possible mechanisms underlying the enhanced response of the NiO nanosheet sensor and the fast response of the MnO_2_@NiO nanosheet sensor toward AM gas. The mechanism underlying the response of semiconductor gas sensors is related to the adsorption of oxygen on the surface of the metal oxide and the subsequent reaction between the adsorbed oxygen and the target gas, resulting in a change in resistance. The same mechanism can also be used to explain the response of NiO and MnO_2_@NiO sensors toward AM gas. The mechanisms of gas sensing in the NiO and MnO_2_@NiO nanosheets are illustrated in the schematic and band diagrams shown in **Figure** **9**.

#### 2.6.1 NiO Nanosheet Sensor

When the gas sensors are exposed to ambient air, oxygen molecules adsorb onto the sensor surface and interact with the metal oxide to form chemisorbed oxygen species (O_2_^−^, O^−^, or O^2−^) depending on the operating temperature.^[^ ^65^ ^]^ As a p‐type semiconductor, NiO provides electrons to oxygen ions, and these charged oxygen species form a hole accumulation layer, also called the depletion region, thereby reducing the sensor resistance. After the NiO sensors are stabilized in ambient air, they are exposed to AM gas (reducing gas). The gas molecules are oxidized, which then react with the oxygen chemisorbed and deficient on the sensor surface. The gas products after the reaction between the AM and oxygen ions were analyzed by GC/MS. First, the AM gas was detected at room temperature before oxidized by NiO gas sensor. As shown the red curve in Figure S12 (Supporting Information), the elution peak at the retention time (Rt) of 6.012 min observed from the gas chromatogram was AM gas, and its mass spectrum was shown in Figure S13a (Supporting Information), there is a strong molecular proton peak at *m*/*z* = 74, which can be speculated to be AM gas. There is another elution peak at Rt = 7.881 min in the gas chromatogram, inferring from its mass spectrum (Figure S13b, Supporting Information), it is the sulfide, allyl methyl gas, which is the main impurity of the AM chemical (> 70%). Then the target gas was through the 225 °C NiO gas sensor and was oxidized, the final decomposition products were further analyzed by GC/MS. The generated species had no elution peak at Rt = 6.012 min, it indicated that the AM gas completely reacted when it is in contact with the NiO gas sensor. The elution peak at Rt = 7.516 min, which mass spectrum (Figure S13e, Supporting Information) was corresponding to benzene was observed. It can be speculated that the AM gas was dehydrogenated and cyclized to form benzene, sulfur dioxide (Figure S13d, Supporting Information) and water. Moreover, when sulfide, allyl methyl also went through the NiO gas sensor at 225 °C, peak area only left one tenth of that before the reaction, which is the evidence of the reaction of sulfide, allyl methyl, the decomposition products were thiophene (Figure S13f, Supporting Information) and water. A very small fraction of the reaction yields tetrahydrofuran (Figure S13c, Supporting Information). They mainly react according to the following equation:

(4) $$2C_{3}H_{6}S+7O^{-}\to C_{6}H_{6}+2{\mathrm{SO}}_{2}+3H_{2}O$$

(5) $$4C_{3}H_{6}S+7O_{2}^{-}\to 2C_{6}H_{6}+4{\mathrm{SO}}_{2}+6H_{2}O$$

(6) $$C_{4}H_{8}S+2O^{-}\to C_{4}H_{4}S+2H_{2}O$$

(7) $$C_{4}H_{8}S+O_{2}^{-}\to C_{4}H_{4}S+2H_{2}O$$

When the above reactions occur, a large number of electrons are released back into the accumulation layer of the sensor surface, which decreases the hole carrier concentration and increases the observed resistance. The sensing mechanism is based on the change in resistance of the sensor surface by the adsorption and desorption of oxygen molecules.

#### 2.6.2 MnO_2_@NiO Nanosheet Sensor

The MnO_2_@NiO composite nanosheets are a typical p–p heterojunction MOS in which the holes are the charge carriers. The energy band diagram of the NiO and MnO_2_ heterojunctions, shown in Figure 9, demonstrates that band bending occurs before and after contact. The heterojunction between NiO and MnO_2_ plays an important role in enhancing the gas response. As reported, the bandgaps of NiO and MnO_2_ are respectively 3.37^[^ ^66^ ^]^ and 1.53 eV.^[^ ^67^ ^]^ Because the valence band energy of NiO is lower than that of MnO_2_, the charge carriers are transferred from NiO to MnO_2_ to equalize the Fermi levels, and a potential barrier is established at the interface of the NiO and MnO_2_ nanosheets.^[^ ^66^, ^68^ ^]^ Thus, the resistance of the NiO/MnO_2_ composite is higher than that of pure NiO. At the thermal equilibrium state, this leads to a hole depletion layer (HDL) on the NiO side and a hole accumulation layer (HAL) on the MnO_2_ side. A schematic diagram of the above mechanism is shown in Figure 9. When the sensor is exposed to AM, the adsorbed oxygen ions react with AM to release electrons back to the surface, thus increasing the resistance of the sensor. Near the heterojunction interface, the released electrons disrupt the dynamic carrier balance between NiO and MnO_2_, and the holes transfer from NiO to MnO_2_. This process narrows the thickness of the HAL on the NiO surface, especially in the area near the heterojunction interface, thus increasing the variation in resistance and the sensor response.^[^ ^69^ ^]^ Thus, the response of the MnO_2_@NiO nanosheets is promoted. As a result, the response is higher, and the detection limit is lower for AM.

Multiple factors contribute to an enhanced performance of the sensor, and, in our case, the p–p heterojunction effect should be considered in conjunction with many influencing factors. The NiO‐nanosheet sensor has a higher response than the MnO_2_@NiO nanosheet sensor, which may be attributed to the following three factors. The surface morphology was first considered. The NiO nanosheets support a porous structure (Figure 2a; SEM images), which effectively increases the surface area and improves gas sensing performance. A nanosheet structure with high porosity provides abundant active sites for chemisorption and reactions with AM, thus increasing the adsorption capacity. Moreover, this structure facilitates gas transfer across its surface by shortening the diffusion distance, which is crucial for interactions between the sensing material and target gas. This becomes the deciding factor in determining the sensitivity of the gas sensor. Therefore, the nanosheets of NiO promote excellent sensing properties.

Moreover, nanosheet structures with high porosity are favorable for the adsorption of oxygen molecules (see the XPS results in Section 3.1). XPS spectra were measured to further investigate the sensing mechanism of the NiO and MnO_2_@NiO gas sensors. The O 1s XPS spectra of NiO and MnO_2_@NiO were analyzed in detail, and the specific binding energy of O_L_, O_V_, and O_C_ are summarized in **Table** **2**. Generally, O_V_ can provide active sites for the adsorption and reactions of the target gas on the surface of the sensing materials, while the O_C_ chemisorbed oxygen species formed on the surface can participate in the sensing reaction.^[^ ^68^ ^]^ Thus, increasing the O_V_ and O_C_ contents can greatly improve the gas sensing response. In the MnO_2_@NiO nanosheets, the O_L_ content is high (>50%). With the formation of MnO_2_@NiO heterojunctions, the O_L_ content becomes higher than that in the pure NiO nanosheets, thus decreasing the sensing performance.

**Table 2:** Analysis of XPS spectra of NiO and MnO_2_@NiO nanosheets (O 1s peak)

	Binding energy [eV]			Area [%]		
Sample	O_C_	O_V_	O_L_	O_C_	O_V_	O_L_
NiO	531.47	529.63	528.78	57.83	30.94	11.23
MnO_2_@NiO	533.53	531.51	529.65	6.15	41.54	52.30

Furthermore, the response of the NiO sensor is significantly higher than that of the MnO_2_@NiO sensor for all AM concentrations because of the structural advantage of the former (as shown in Figure 2a) and low activation energy. The degrees of each sensitization mechanism can be interpreted by the activation energy of gas sensor since each sensitization involves activation changes. The activation energy can be evaluated from the temperature dependence of the resistance using the Arrhenius equation.^[^ ^70^, ^71^ ^]^

**Figure 10:**
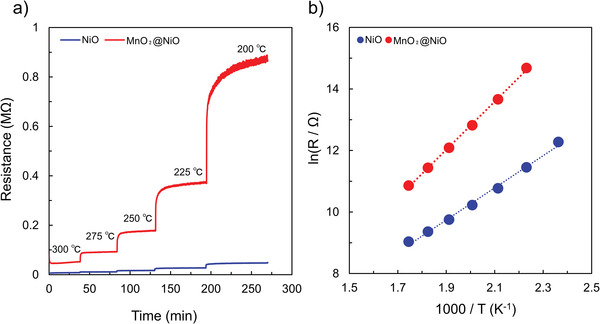
a) Resistance variations of NiO and MnO_2_@NiO in air versus temperature. b) Dependence of the natural logarithm of the resistance on inverse temperature for the NiO and MnO_2_@NiO gas sensors.

The gas sensing mechanism and the cause of the higher response and reduced operating temperature of the NiO sensor were investigated. As shown in **Figure** **10**a, the resistance of the NiO and MnO_2_@NiO gas sensors in air varied with temperature.

In the temperature range of 175–300 °C, the resistance increased rapidly. In the lower temperature range, the resistance was high. With increasing temperature, the resistance became smaller. The variation in the bulk resistance of the material was found to be essentially governed by the temperature, because both the charge of the surface species (O_2_, O_2_^−^, O^−^, and O^2−^) and their coverage could alter in the temperature‐changing process, which in turn affects the surface resistance (originating from the HAL). Therefore, the working temperature plays a crucial role in determining the sensor response. Additionally, the activation energy, which reflects the surface reactivity of the materials was calculated. The natural logarithm of resistance versus the reciprocal of absolute temperature was plotted (Figure 10b), revealing a linear relationship between the two parameters. The slope of the curve can be used to determine the activation energy (*E*_a_) using the Arrhenius Equation (8):

(8) $$R=R_{0}e^{\frac{E_{a}}{K_{B}T}}$$

where *R* is the resistance, *R*_0_ is a constant, *E*_a_ is the activation energy, *K*_B_ is the Boltzmann constant, and *T* is the absolute temperature of the material. From the slope of the linear zone, the calculated activation energy for NiO was 0.45 eV, which was lower than the activation energy for MnO_2_@NiO (≈0.67 eV). Therefore, the lower activation energy of the intrinsic conduction contributes to the enhanced gas sensing properties of the NiO nanosheets at the reduced operating temperature of 225 °C. According to the Arrhenius plots, these two materials exhibited significant resistance changes. The experimental results matched the numerical model of p‐type resistive gas sensors developed by Bejaoui et al.^[^ ^72^ ^]^

The effect of MnO_2_ and the surface morphology of the nanosheets induced by the solution growth treatment can be explained as follows. The MnO_2_@NiO composite can form p–p heterojunctions to improve the gas response. Moreover, during the growth of MnO_2_, the size of the nanosheets becomes smaller. These factors positively influence the sensor performance, which leads to a faster response for the MnO_2_@NiO sensor. However, during the growth of MnO_2_ on the surface of the NiO nanosheets, the pores on the NiO nanosheets disappear. Therefore, the surface area decreases and gas diffusion into the sensor is obstructed. This may be one of the important reasons for the reduced sensitivity of the MnO_2_@NiO sensor.

In the case of MOS gas sensors, a surface‐controlled process is responsible for the response. The performances of the NiO and MnO_2_@NiO sensors obtained in this study are the result of various complex factors interacting with each other. This is the first time that the AM gas has been detected by MOS gas sensor, and high concentrations of this gas can be studied under the current experimental conditions. The MnO_2_@NiO sensor is prospected to perform well for low concentrations of the AM gas, i.e., fast, high response, and good selectivity. Such speculate is based on the LOD values in this study. The calculated LOD values of the NiO and MnO_2_@NiO sensors are similar, indicating that both can detect AM gas at low concentrations. A detailed investigation of the sensing performance and mechanism of NiO and MnO_2_@NiO nanosheets to detect AM vapor is currently underway in our group.

## 3 Conclusion

In summary, we developed simple chemiresistor‐type gas sensors based on porous NiO nanosheets and NiO modified by MnO_2_. The sensors exhibited high sensitivity, good selectivity, fast response, and good reversibility toward the AM gas, which is very important because AM is a component of the chemical signature of psychological stress. Pure NiO and MnO_2_‐coated NiO nanosheets were directly synthesized onto interdigitated electrodes via a solvothermal method and subsequently calcined at 400 °C. These films possessed excellent sensing properties; specifically, the NiO sensor detected 12 ppm of AM (SNR > 5.6 × 10^4^) at 225 °C with high response as 41.18, it was approximately four times higher than that of the MnO_2_@NiO sensor. However, the response time of the MnO_2_@NiO sensor was more than four times faster than that of the NiO sensor at 40 ppm of AM. The high response of the NiO sensor was attributed to the nanostructured porous morphology, which facilitated rapid gas transport and increased the adsorbed oxygen content on the surface of the nanosheets. Both factors can enhance the response of the materials. The lower activation energy also promotes the reactions between the target gas and the NiO nanosheets to enhance the response value of the gas sensor. The faster response time of the MnO_2_@NiO sensor is the result of the p–p heterojunction of NiO and MnO_2_ and the reduced size of the nanosheets. Low theoretical LODs (15 ppb and 32 ppb) were obtained for AM detection. Most importantly, the NiO nanosheet films exhibited outstanding selectivity over various breath‐ and indoor‐relevant confounders (ethanol, acetone, isoprene, toluene, ammonia, hydrogen and nitrogen dioxide). The response for AM was 37 times higher than that for hydrogen gas and at least 17 times higher than that for acetone gas. Therefore, the developed sensors are highly selective for AM. The NiO porous nanosheets and the MnO_2_‐modified NiO nanosheets were synthesized in one step; this facile process is cost‐effective and therefore attractive for gas sensing applications.

Psychological stress is universal in our lives. Numerous studies indicate that psychological stress has a negative impact on blood pressure, heart disease, and physical health. It can also cause memory decline, cognitive failure, and even mental illnesses, such as depression and anxiety.^[^ ^12^, ^73^, ^74^ ^]^ Gas sensors based on MOS can rapidly and non‐invasively provide information on the status and progression of psychological stress in people. Thus, people can easily identify psychological stress and concurrently seek treatment to avoid various health problems caused by psychological stress. Ultimately, this may dramatically reduce medical costs and improve the quality of life. Further studies are required to achieve AM detection at lower concentrations and under different atmospheres, which is necessary for the analysis of exhaled breath.

## 4 Experimental Section

### Experimental Details

Nickel chloride hexahydrate (NiCl_2_·6H_2_O), urea (CH₄N₂O), and potassium permanganate (KMnO_4_) were purchased from Fujifilm Wako Pure Chemical Corporation (Japan). All the chemicals were of high purity and did not require further purification. Distilled water was used for the experiments.

### Preparation of Ni(OH)_2_ Precursor

The alumina chips were used as the sensor substrate for the in situ growth of the NiO nanosheets. The Pt interdigitated electrodes and microheater were fabricated respectively on the top and bottom sides of the alumina substrate by screen printing technologies. The alumina chips were irradiated with vacuum ultraviolet light for 20 min to obtain a clean and uniform wetting substrate surface. Ni(OH)_2_ nanosheets were synthesized onto the alumina chips using a solvothermal method. Briefly, NiCl_2_·6H_2_O (0.2375 g, 1 mmol) and CH₄N₂O (0.3 g, 5 mmol) were dissolved in 80 mL of a mixed solution of ethanol and water with a volume ratio of 1:3. The mixture was stirred constantly and vigorously for 30 min to form a clear green solution. Pretreated sensor chips were immersed into a 250 mL polypropylene cup filled with the prepared solution, and the polypropylene cup was heated in an oven at 84 °C for 6 h for the growth of Ni(OH)_2_ nanosheets on the alumina chips, and the alumina chip was found to be uniformly covered by the as‐synthesized green Ni(OH)_2_. The obtained Ni(OH)_2_ sensor chips were washed several times with distilled water and dried at 60 °C for 15 h. Ni(OH)_2_ synthesized on the alumina substrate can be directly used as sensor chips after calcination or as precursors for the synthesis of MnO_2_ nanosheets. Concurrently, the green powders suspended in the cup were collected by centrifugation and post‐treated with the same process, it was used as the precursor to the synthesis powder of MnO_2_@NiO nanosheets.

### MnO_2_ Coating

KMnO_4_ (0.2 g) was dissolved in 100 mL of water. The as‐prepared Ni(OH)_2_ sensor chips were placed in the polypropylene cup, and the reaction was carried out at 65 °C for 2 h. The sensor chips, covered with the nanosheets, were washed repeatedly with distilled water (to remove ions and molecules from their surface) and then dried at room temperature overnight. Concurrently, MnO_2_@NiO powder was synthesized by using the Ni(OH)_2_ powder as a precursor through the same synthesis method and posttreated process. Finally, the dried samples were annealed in a furnace at 400 °C for 5 h in air with a heating rate of 2 °C min^−1^ to remove the residual solvent and to decompose Ni(OH)_2_, thus transforming the MnO_2_@Ni(OH)_2_ precursor nanosheets into MnO_2_@NiO nanosheets.

### Material Characterization

The morphology of the products was determined using a JEOL JSM‐6335FM FESEM (Japan) operating at 10 kV to obtain top‐view and cross‐sectional images. The crystalline structure was determined using a Rigaku XRD (Japan) operating at 40 kV and 30 mA with Cu K*α* radiation. XRD patterns were collected at a scanning speed of 0.01° min^−1^ over the 2*θ* range of 20°–150°. The elemental composition and surface chemical state of the samples were determined using a Shimadzu ESCA‐3400 XPS (Japan). All the binding energies were calibrated to the C 1s peak at 284.6 eV of the surface adventitious carbon. Thermogravimetric analysis (TGA, MTC1000SA, Bruker, USA) was used to investigate the thermal behavior of the materials.

### Gas Sensing Measurements

The sensing performance of the NiO and MnO_2_@NiO films was evaluated using a gas multisensor system. The sample was placed in an airtight stainless‐steel chamber to continuously monitor the film resistance between the interdigitated electrodes. The working temperature was set by applying a DC voltage (Texio, pw8‐3AQP, Japan) to the substrate heater according to the linear relationship between the applied voltage and the temperature of the gas sensor, and this linear relationship was measured using a thermal camera (FLIR, Japan; Figure S14, Supporting Information).

The reference gas mixtures were prepared using a mixing setup (Holiba, MU‐3609, Japan). Briefly, the analyte gases (i.e., ethanol, acetone, isoprene, toluene, ammonia, hydrogen, and nitrogen dioxide) were supplied from cylinders (25 ppm in synthetic air) and infused into dry synthetic air with calibrated mass flow controllers. There were no commercially available cylinders for AM. Therefore, we used the gas generation system (Gastec, PD‐18, Japan) to produce the gas from the liquid at 35 °C. AM (C_3_H_6_S) was purchased from Tokyo Chemical Industry Co., Ltd. (Japan, >70%). The concentration of AM gas was 75 ppm when the N_2_ flow rate was 80 mL min^−1^; this amount was calculated using the calibration curve. During practical testing, the concentration of the target gas was changed by diluting with synthetic air. Dry synthetic air was used to dilute the test gas to the desired concentration and also to clean the gas chamber before and after test‐gas exposure. Prior to the measurements, the sensors were heated to the desired operating temperature for 2 h in dry ambient air for thermal stabilization. Gas sensing measurements for various AM vapor concentrations (12–40 ppm) diluted with air were performed at the optimized operating temperatures. The gas chromatograph used during the analysis was an Agilent 6890 equipped with an Agilent 5973 mass spectrometer detector (MSD). GC/MS analysis was combined with a thermal desorption system (TDS) for injection of gases adsorbed on fibers.

The sensing response (*S*) was determined using the variation in resistance according to equation *R  =  R_g_/R_a_* for oxidizing gases such as nitrogen dioxide; and inverse for reducing gases (*R  =  R_a_/R_g_*). Where *R*_g_ and *R*_a_ are the film resistances after and before test‐gas exposure, respectively. The response time is defined as the time required for the variation in resistance to reach 90% after a test gas was injected, while the recovery time is the time required for the sensor to return to 90% above the original resistance in air.

## Conflict of Interest

The authors declare no conflict of interest.

## Supporting information

Supporting Information

## Data Availability

The data that support the findings of this study are available from the corresponding author upon reasonable request.
