# Supplementary material for: Highly Sensitive and Selective Gas Sensors Based on NiO/MnO2@NiO Nanosheets to Detect Allyl Mercaptan Gas Released by Humans under Psychological Stress
Source: Adv Sci (Weinh). 2022 Jul 15;9(27):2202442. doi: 10.1002/advs.202202442 (PMC9507369; doi:10.1002/advs.202202442)
Supplement: Supplementary file 1 — Supporting Information [file ADVS-9-2202442-s001.pdf]

Supporting information

Highly Sensitive and Selective Gas Sensors Based on NiO/MnO_2@NiO Nanosheets to Detect Allyl Mercaptan Gas Released by Humans under Psychological Stress

*Chunyan Li, Pil Gyu Choi, Yoshitake Masuda**

C. Li, P. G. Choi, Y. Masuda National Institute of Advanced Industrial Science and Technology (AIST), Nagoya 463-8560, Japan.
Email: masuda-y@aist.go.jp

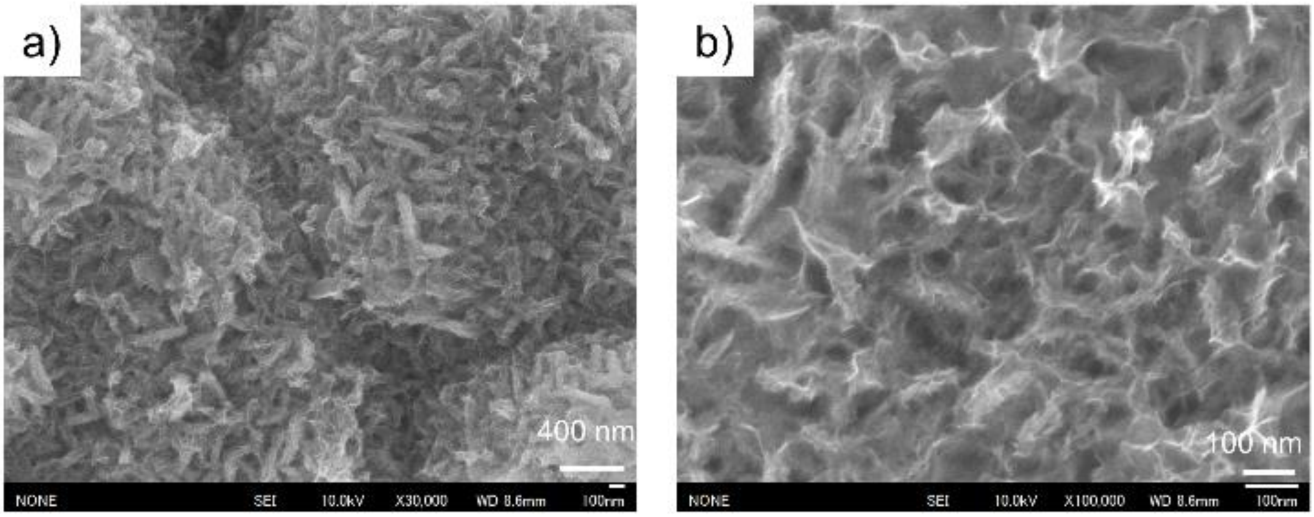

Figure S1. SEM images of a) MnO_2@NiO nanosheets for a synthesis time of 24 h. b) Magnified image of the MnO_2@NiO nanosheets for a synthesis time of 24 h.

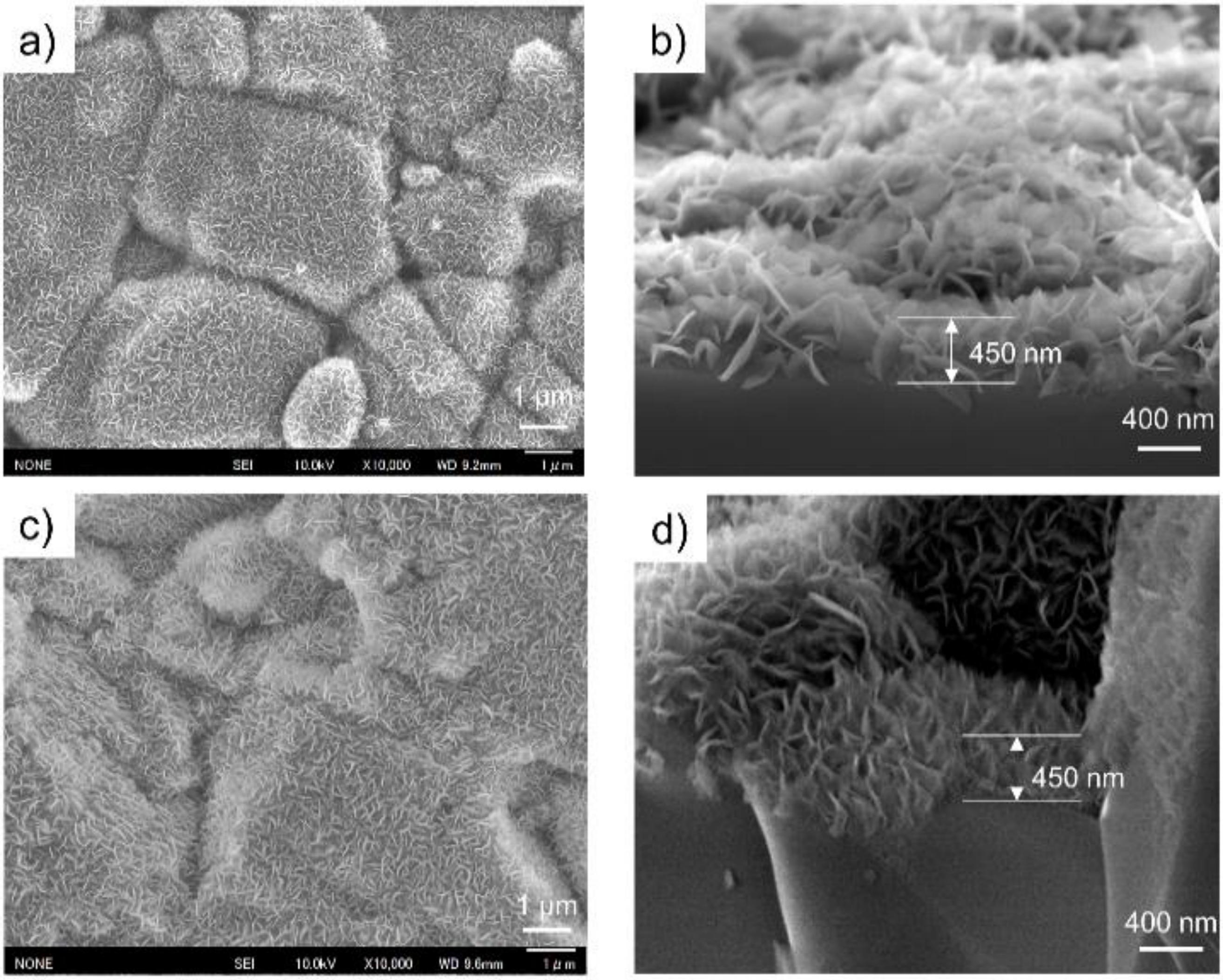

Figure S2. a) Top-view and b) cross-sectional SEM images of NiO. c) Top-view and d) cross-sectional SEM images of MnO_2@NiO.

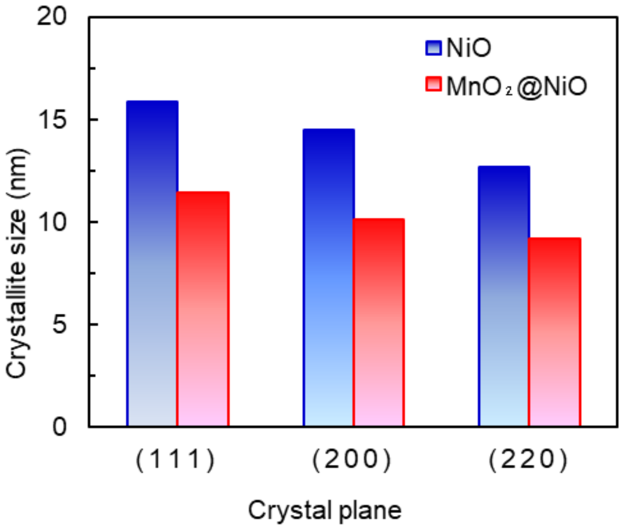

Figure S3. Crystallite size of NiO and MnO_2@NiO calculated using the Scherrer equation.

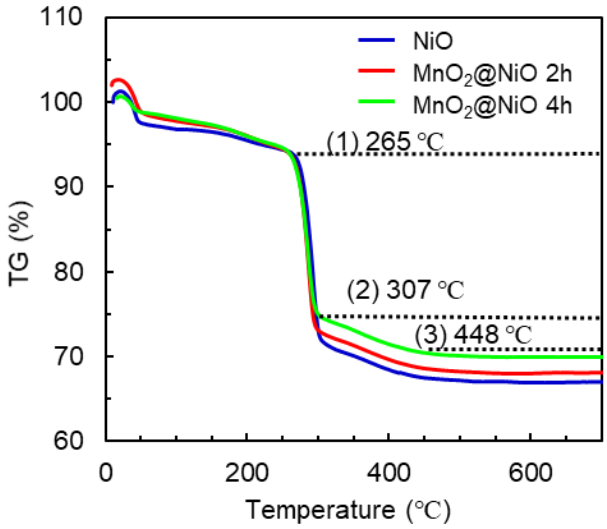

Figure S4. TG curve of NiO and MnO_2@NiO (synthesis time of 2 h and 4 h, respectively).

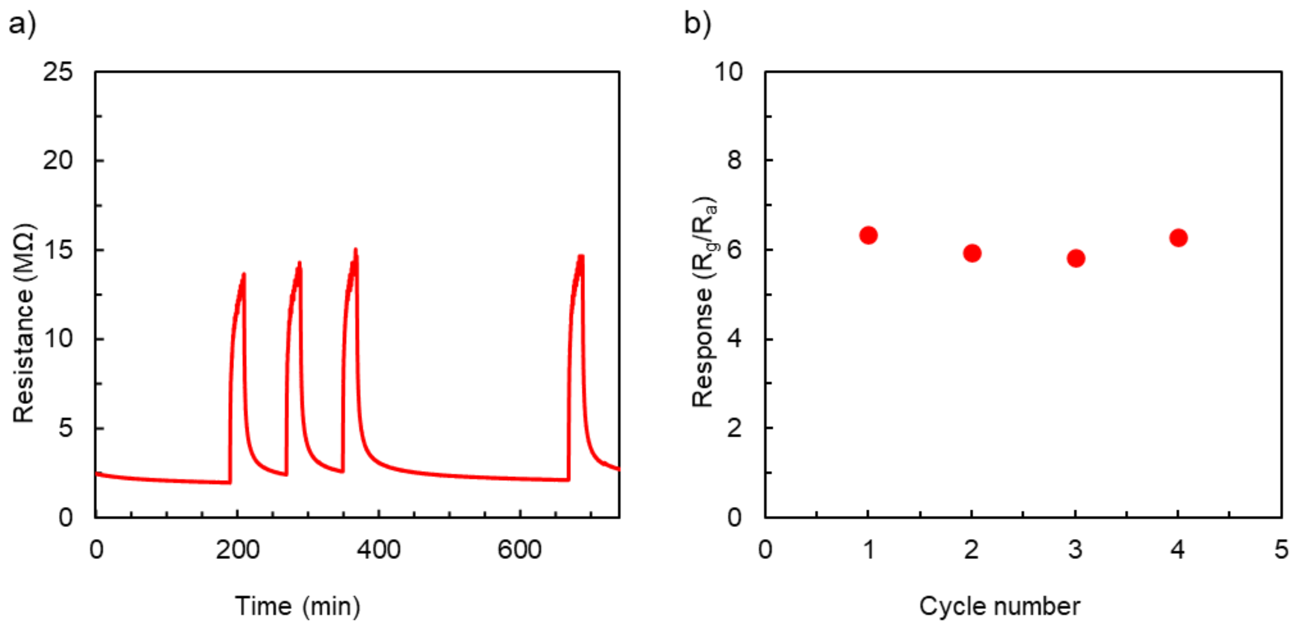

Figure S5. a, b) Dynamic and quantitative responses of the MnO_2@NiO sensor exposed to 12 ppm of allyl mercaptan for four cycles.

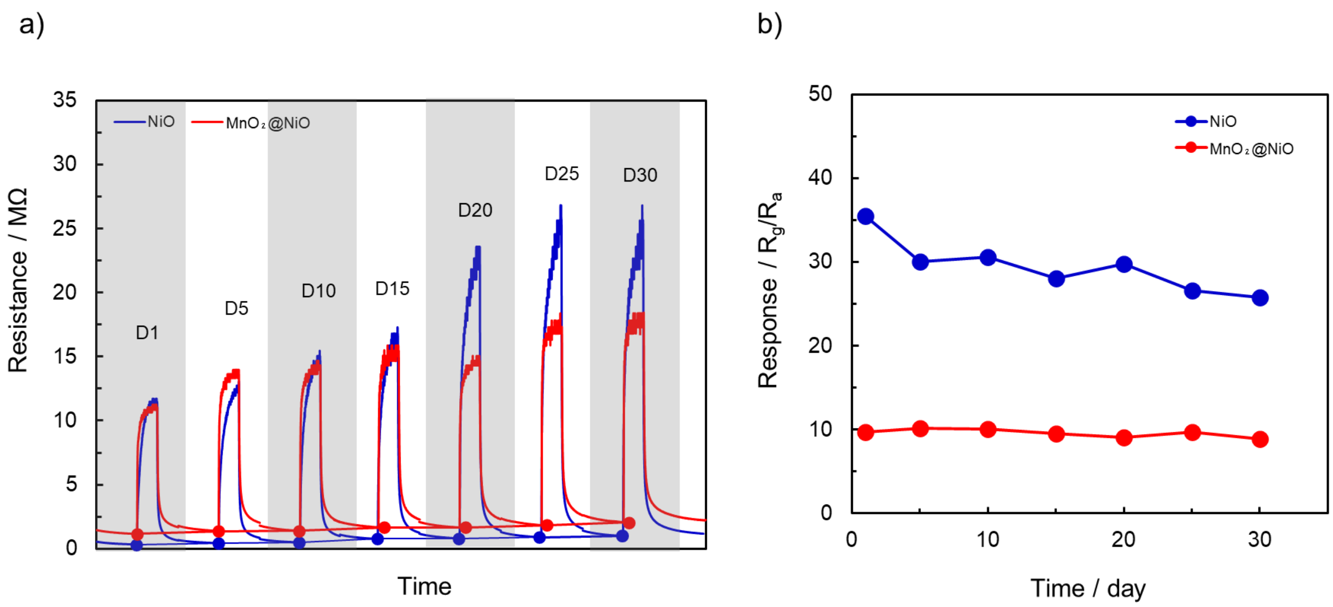

Figure S6. Long-term stability of the gas sensors based on NiO and MnO_2@NiO exposed to 20 ppm Allyl mercaptan within 30 days: a) Dynamic curves and b) quantitative responses.

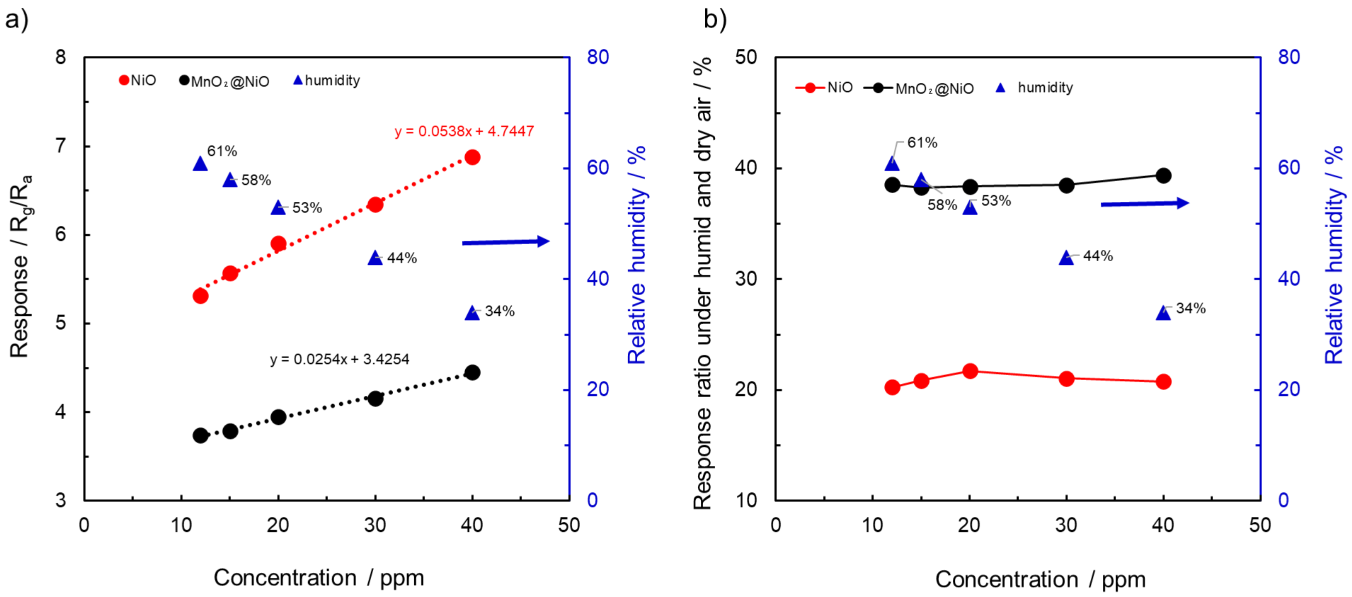

Figure S7. a) Response of the sensors based on NiO and MnO_2@NiO exposed to relative humidity from 34% to 61% and b) the response ratio under humid air and dry air of NiO and MnO_2@NiO sensors in the different relative humidity.

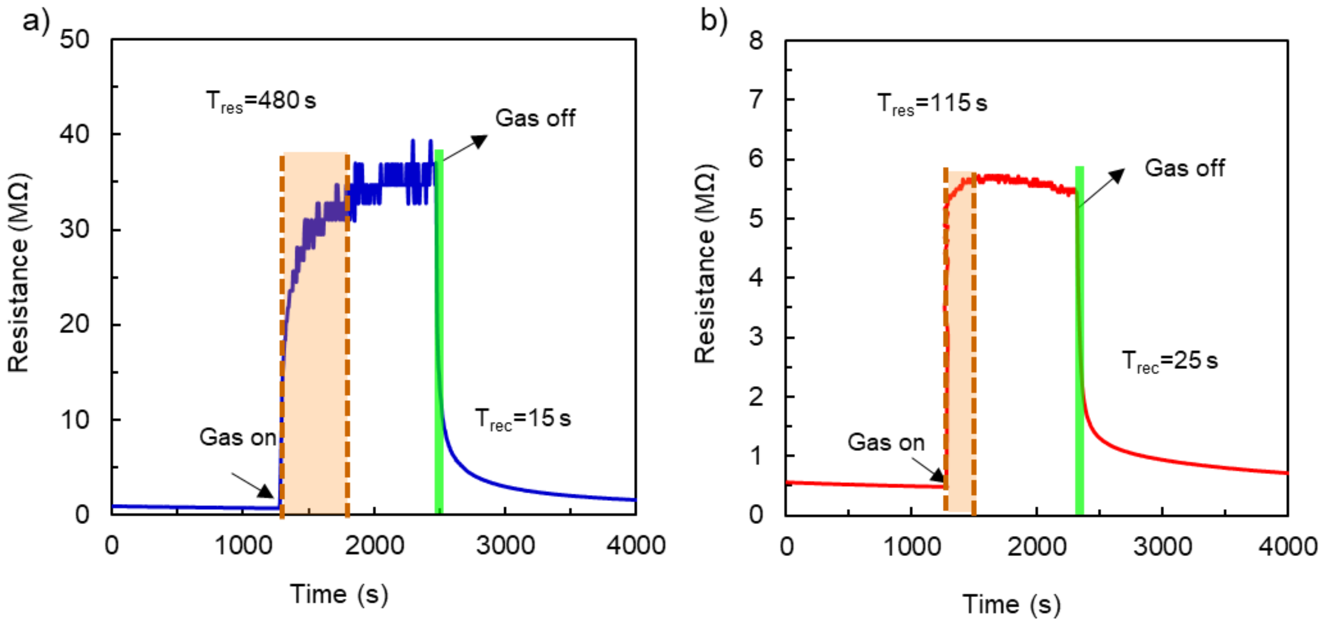

Figure S8. Response transients of the a) NiO and b) MnO_2@NiO gas sensors exposed to 40 ppm of allyl mercaptan gas.

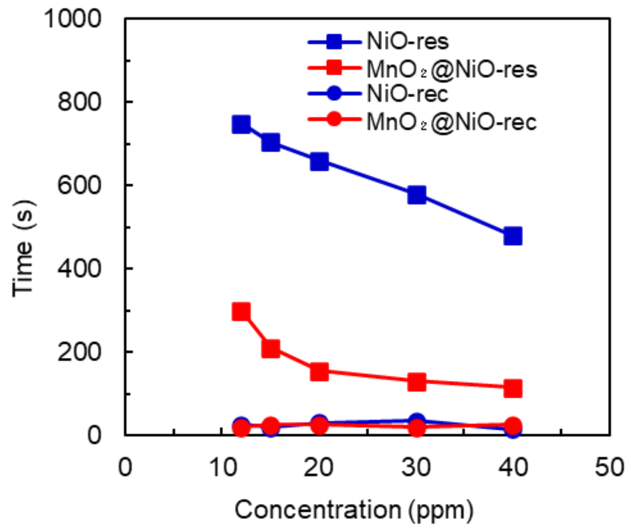

Figure S9. Response (res) and recovery (rec) times of the NiO and MnO_2@NiO sensors exposed to different concentrations of allyl mercaptan.

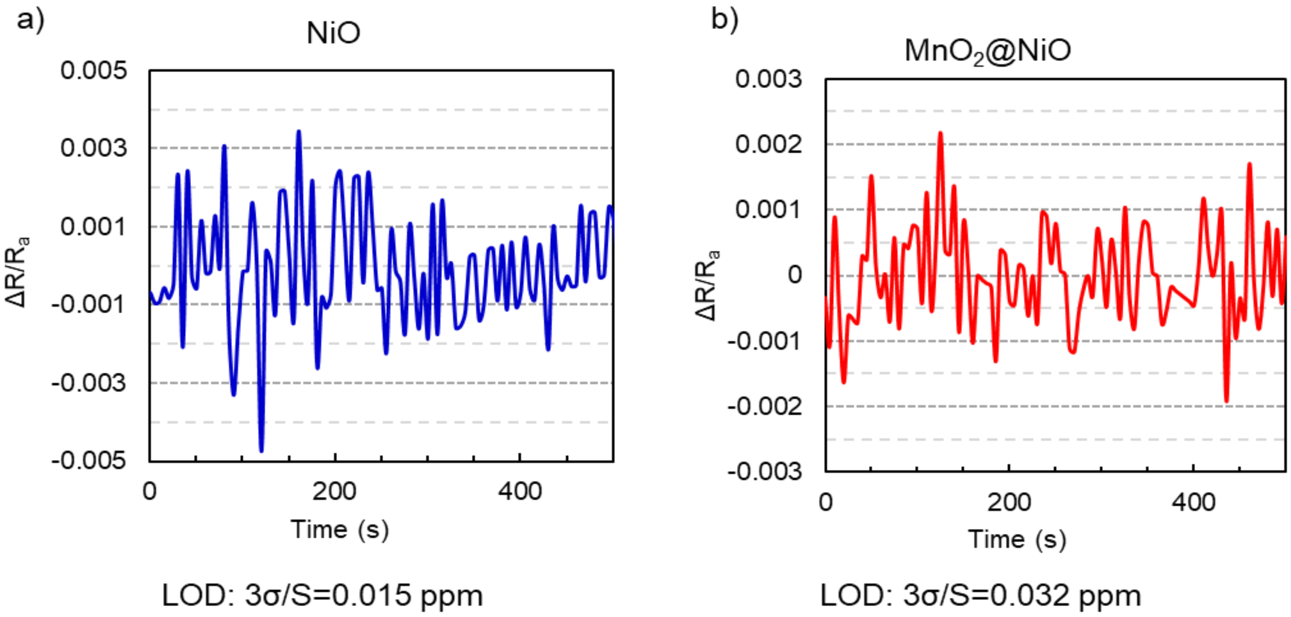

Figure S10. Plots of fitted responses of the a) NiO and b) MnO_2@NiO sensors as a function of time at the baseline before allyl mercaptan exposure.

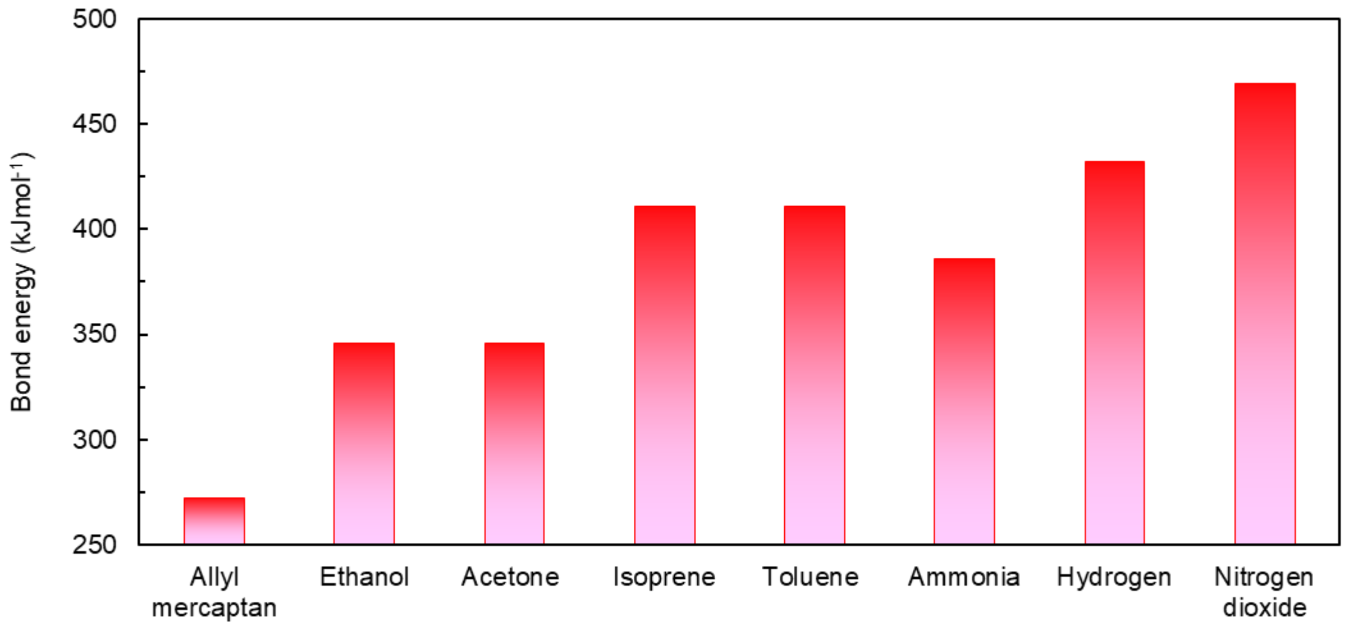

Figure S11. Bond energies for allyl mercaptan, ethanol, acetone, isoprene, toluene, ammonia, hydrogen gas, and nitrogen dioxide. (A low bond energy leads to facile bond cleavage.)

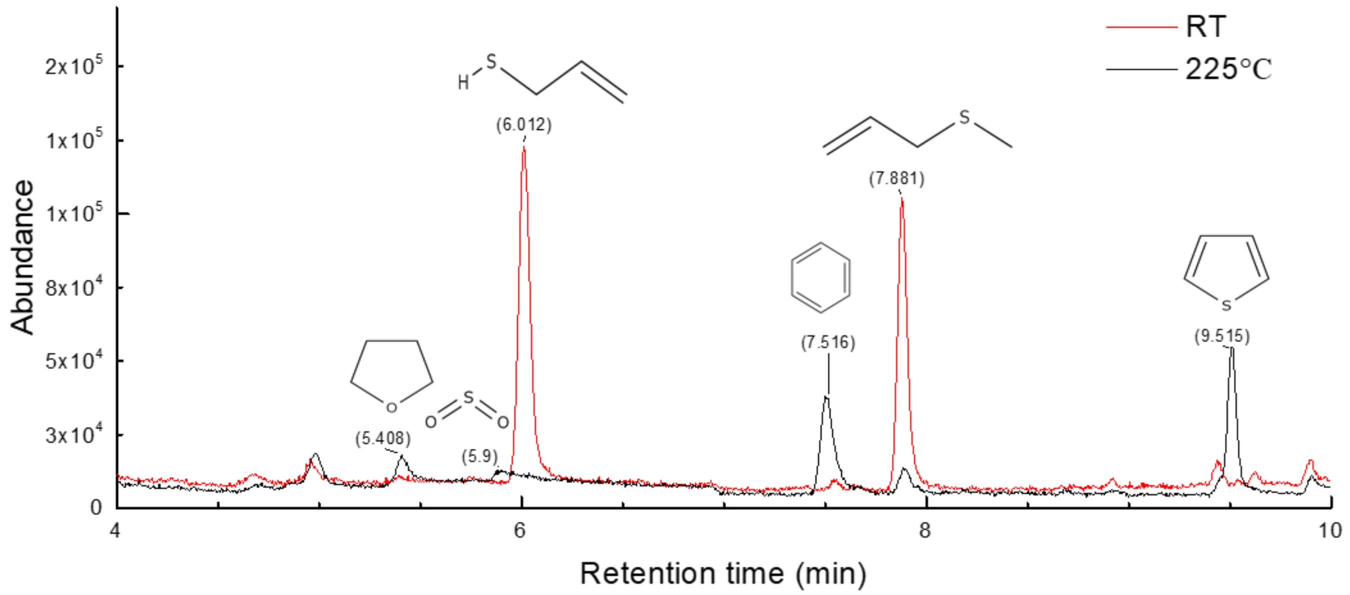

Figure S12. Gas chromatogram of the allyl mercaptan gas at room temperature (red line) and after oxidized at 225 °C by NiO gas sensor (black line).

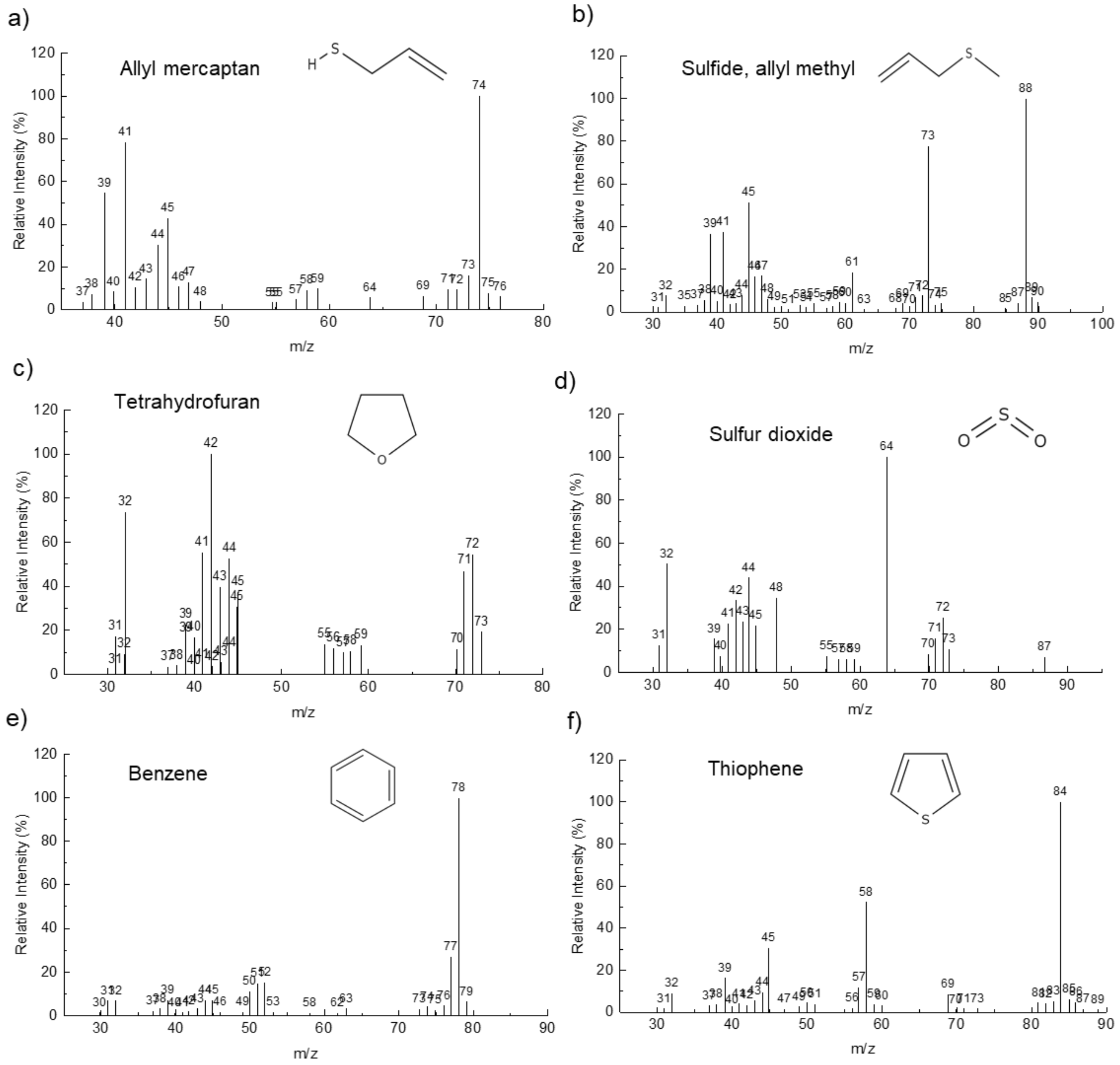

Figure S13. Mass spectra of a) allyl mercaptan, b) sulfide, allyl methyl, c) tetrahydrofuran, d) sulfur dioxide, e) benzene and f) thiophene.

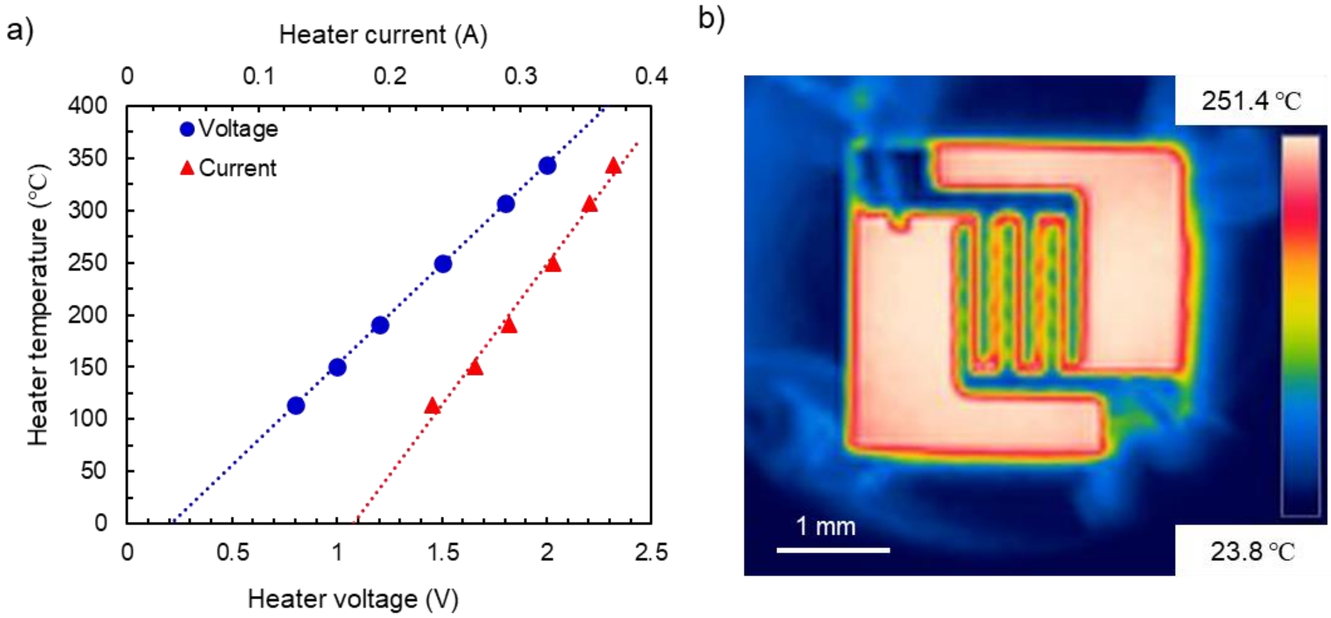

Figure S14. a) Temperature of the substrate as a function of the DC voltage and current applied to the microheater. b) Optical image captured by an infrared camera showing the temperature of substrate (251 °C).
